# Identification of druggable targets in melanoma by multi-omics Mendelian randomization integrated with transcriptomic and spatial analysis

**DOI:** 10.3389/fgene.2025.1657356

**Published:** 2025-10-27

**Authors:** Jiahua Xing, Mingyong Yang, Muzi Chen, Ran Tao

**Affiliations:** ^1^ Plastic Surgery Hospital, Chinese Academy of Medical Sciences and Peking Union Medical College, Beijing, China; ^2^ Department of Plastic and Reconstructive Surgery, The First Medical Center, Chinese PLA General Hospital, Beijing, China

**Keywords:** melanoma, Mendelian randomization, multi-omics, drug repositioning, Bioinforamtics

## Abstract

**Background:**

Cutaneous melanoma (CM) is a highly lethal skin tumor. Some patients respond poorly to existing therapies, and developing new targeted therapies remains challenging.

**Methods:**

We combined the results of eQTLs, pQTLs, and genome-wide association study (GWAS) to identify potential causal effects of two target genes on CM, based on multi-omics Mendelian randomization (MR). Sensitivity analysis, co-localization analysis, and inverse MR analysis were also employed to verify the robustness of this causal relationship. Multi-omics data were then applied to explore the expression patterns of immune infiltration of the target genes and construct nomogram models.

**Results:**

The results showed that the gene prediction levels of EPS15L1 and HGS were associated with an increased risk of CM. Co-localization analysis revealed significant horizontal pleiotropy of the target gene, and reverse MR showed unidirectional causality of the targets. Multi-omics analysis comprehensively demonstrated the expression regulation pattern of the target genes in the CM immune-environment and identified interactions between EPS15L1 (Q9UBC2) and HGS (O14964) and doxorubicin, demonstrating the potential for drug application. The validity of the targets was further verified by molecular biology experiments.

**Conclusion:**

This study provides robust genetic and therapeutic evidence for targeting EPS15L1 and HGS in CM treatment.

## Introduction

Cutaneous melanoma (CM), which is a malignant tumor that originates in melanocytes, continues to increase in incidence worldwide (Seta et al., 2025). According to the 2022 global cancer statistics report, there will be around 331,647 new cases of cancer and 58,645 cancer-related deaths (Bray et al., 2024). The surgical excision of *in situ* CM is an effective treatment, with a favorable prognosis. However, the invasion and metastasis of CM can directly affect the disease stage and significantly influence patient survival. The prevention and treatment of CM continue to present significant challenges (Long et al., 2023).

Recent years have seen a rapid development of high-throughput sequencing technology, which has created new opportunities for targeted therapeutic research in CM (Millet et al., 2017). Genome-wide association studies (GWAS) have successfully identified several single nucleotide polymorphisms (SNPs) that are significantly associated with disease risk through genetic analysis of a large population of CM patients. However, the majority of these genetic variants are located in the distal regulatory regions of the gene regulatory network, which is a considerable distance from the downstream molecular targets that can be directly intervened. This limits their clinical translational value (Finan et al., 2017).

Research has indicated that approximately 40% of the protein-coding genes present within the human genome have been identified as druggable, thus providing significant insights into the development of targeted medicinal treatments (Chen et al., 2023). However, traditional observational studies are encumbered by significant limitations in their capacity to identify potential therapeutic targets, including the interference of environmental confounders (like history of UV exposure, host immune status) and the potential for bidirectional causality between protein expression levels and disease phenotypes. Mendelian randomization (MR) is an emerging causal inference method that effectively circumvents the problem of confounding bias in traditional studies by utilizing genetic variation as an instrumental variable (IVs) (Sekula et al., 2016). The integration of GWAS data with multi-omics data, including protein quantitative trait loci (pQTLs) and expression QTLs (eQTLs), enables a systematic assessment of the causal relationship between genetically predicted alterations in the expression levels of specific proteins or genes and CM risk (Rasooly et al., 2023) (Wang et al., 2024).

The objective of this study was to identify causal biomarkers and potential therapeutic targets for CM by integrating GWAS data and multi-omics data, such as eQTLs and pQTLs. Furthermore, the causal relationship between the targets and CM was to be confirmed through sensitivity analysis, co-localization analysis and inverse MR analysis. Subsequent studies have corroborated the function of the targets in the immune microenvironment and tumorigenesis progression of CM through further analysis at the transcriptome level. Moreover, molecular docking was employed to assess drug repurposing, thereby providing novel insights into the target therapy of CM.

## Materials and methods

### Data acquisition

In order to conduct a single-cell transcriptome analysis, data from nine tumor samples from GSE207592 were downloaded from the NCBI GEO public database (https://www.ncbi.nlm.nih.gov/geo/info/datasets.html). Concurrently, data from four tumor samples from GSE238004 were downloaded for spatial transcriptome analysis. Expression data from the raw processed SKCM were downloaded from the TCGA database (https://portal.gdc.cancer.gov/), and a total of 473 patients were included in the expression profile data, and 556 cases of raw processed expression data were downloaded from the GTEX database as normal controls for CM. The exposure data that was selected comprised eQTL data obtained from the eQTLGen consortium database (https://www.eqtlgen.org), while the pQTL data was obtained from deCODE (https://www.decode.com/summarydata/). The deCODE data set describes the 35,559 Europeans who participated in GWASs, with plasma protein levels measured using 4,907 aptamers. The summary outcome data selected for this study were obtained from the FinnGen biobank database (finngen_R12_C3_MELANOMA_SKIN_EXALLC), and the analysis of CM cases was conducted using their corresponding International statistical classification of diseases (ICDs). Of these, 5,753 cases were classified as CM and 378,749 as normal cases.

### MR analysis of eQTLs and pQTLs

In the eQTL and pQTL analyses, SNPs significantly associated with each gene at the genome-wide significance threshold (*P* < 1 × 10^−8^) were selected as potential instrumental variables (IVs). To minimize confounding bias, we further excluded SNPs associated with known confounders (e.g., UV exposure history, immune status, smoking status) using the PhenoScanner database (screening threshold *P* < 1 × 10^−5^). Linkage disequilibrium (LD) clumping was performed using the 1000 Genomes European reference panel (Phase 3 v5), retaining only SNPs with *R* ^2^ < 0.001 within a 10,000 kb window. The selection of *R* ^2^ < 0.001 and a 10,000 kb window size was based on the following rigorous considerations (Auton et al., 2015; Hemani et al., 2018). First, the stringent *R* ^2^ < 0.001 threshold ensures low correlation between instrumental variables (*r* ^2^ ≈ 0.03), which is more conservative than the commonly used *R* ^2^ < 0.01 standard in GWAS studies, effectively avoiding estimation bias due to linkage disequilibrium, particularly in complex loci such as the MHC region. Second, the 10,000 kb window size was carefully optimized to cover the regulatory scope of most cis-eQTLs (typically located within 1 Mb upstream and downstream of transcription start sites) while avoiding the influence of long-range linkage (e.g., chromatin loop-mediated interactions). Furthermore, this parameter combination aligns with the processing standards of authoritative databases such as deCODE and eQTLGen, ensuring the comparability of our results. We validated the robustness of these parameters through sensitivity analysis. When adjusting the window size to 5,000 kb or 20,000 kb, the core instrumental variable set remained highly consistent (Jaccard similarity coefficient >0.85), and the variation in MR result estimates was less than 10%, indicating that our conclusions are insensitive to parameter selection. The causal effects were primarily estimated using the inverse-variance weighted (IVW) method, supplemented by MR-Egger, weighted median, and weighted mode methods to ensure robustness. For SNPs with only one available statistic, the Wald ratio test was applied. Horizontal pleiotropy was assessed via the MR-Egger intercept test. Sensitivity analyses included leave-one-out validation and consistency checks across methods (e.g., directionally aligned effect estimates and overlapping confidence intervals). All analyses were conducted using the TwoSampleMR package (v0.5.6) in R, leveraging summary-level data from the eQTLGen consortium (whole blood) and the deCODE pQTL database.

### Sensitivity analysis

To comprehensively evaluate the robustness of causal inferences, we implemented multiple sensitivity analyses. First, we assessed horizontal pleiotropy through the intercept test of MR-Egger regression (requiring *P* > 0.05). Second, we verified the consistency of primary results using the weighted median and weighted mode methods (requiring effect directions aligned with IVW and overlapping confidence intervals). Finally, we applied the leave-one-out method to systematically exclude each SNP and recalculate the pooled effect size, evaluating the influence of individual genetic variants on the overall estimates. All analyses were conducted using the TwoSampleMR package (v0.5.6) in R, with multiple testing correction via the Benjamini–Hochberg method (FDR <0.05).

### SMR co-localization analysis

The software tool known as SMR was originally conceived with the intention of implementing the SMR and Heterogeneity in Dependent Instruments (HEIDI) approaches. The utilization of pooled level data was to be employed in order to facilitate an examination of pleiotropic associations between gene expression levels and complex traits of interest (Zhu et al., 2016). We employed the SMR and HEIDI framework to investigate whether genetic associations between gene expression/protein levels and CM risk were driven by shared causal variants. This analysis integrated GWAS summary statistics from FinnGen R12 with eQTL data from the eQTLGen consortium and pQTL data from deCODE. The SMR test (*P* < 0.05) evaluated mediation effects of gene expression/protein levels on disease risk, while the HEIDI test (*P* ≥ 0.05) distinguished true colocalization from linkage disequilibrium artifacts. Specifically, the SMR-HEIDI framework tests whether the effect of a SNP on a phenotype is mediated by gene expression or protein levels, thereby prioritizing potential causal gene targets for GWAS loci. Instrumental SNPs were selected with stringent LD clumping to ensure robustness.

### Immune infiltration

Immune infiltration analysis was performed using the CIBERSORT algorithm (v1.04) to quantify the proportions of 22 immune cell types in the tumor microenvironment. This algorithm employs a support vector regression model to deconvolve the expression matrix of 547 signature genes (LM22 signature matrix), calculating the relative abundance of immune cell subtypes. Correlations between core gene expression and immune cell content were assessed using Spearman’s correlation coefficients.

### Nomogram model construction

The nomogram was constructed based on a multivariate Cox regression model using the rms package (v6.7–0). Regression coefficients were converted into a scoring system, with each variable’s contribution visualized as point values. The total score corresponded to predicted 2-, 3-, and 5-year overall survival rates.

### Single-cell data processing and annotation

Single-cell data processing was performed using the Seurat pipeline (v4.3.0). Quality control excluded cells with UMI counts <200 or >5000, gene counts <500, or mitochondrial gene percentage >10%, with doublets removed by DoubletFinder (v2.0.4). Data normalization employed LogNormalize (scale factor 10,000), followed by highly variable gene selection (2,000 genes) and Z-score standardization (ScaleData). After batch effect correction with Harmony (v1.2.0), dimensionality reduction was conducted via PCA (top 30 PCs) and UMAP (min.dist = 0.3), with clustering using the Louvain algorithm (resolution = 0.8). Cell annotation was completed based on marker gene expression patterns from the CellMarker database and SingleR (v2.4.0).

### Pseudo-time analysis

Pseudotime analysis was performed using Monocle3 (v1.3.4) to construct cell differentiation trajectories. The reverse graph embedding algorithm (DDRTree) projected cells onto a pseudotime axis, and differentially expressed genes along pseudotime were identified (q-value <0.01).

### RCTD reverse convolution

RCTD (Robust cell-type decomposition) is a supervised learning method that decomposes RNA-sequencing mixtures into individual cell types, thereby enabling the assignment of cell types to spatial transcriptome pixels. Spatial transcriptome deconvolution utilized the RCTD algorithm (v2.4.0), which leveraged annotated scRNA-seq reference data to decompose cell type proportions in spatial spots. The dominant cell type per spot was determined via maximum likelihood estimation (confidence >0.7).

### Molecular docking

As indicated by the key genes, the 3D structures of proteins in the alphafold database (https://alphafold.com/) were obtained, and the drug prediction of the key genes was performed in the CTD database (https://www.ctdbase.org/). The related essentials were then obtained, and the structures of drug components were obtained through the PubChem database (https://pubchem.ncbi.nlm.nih.gov/) to obtain the structures of drug components. Using AutoDock Vina software, with a grid box size set to 60 × 60 × 60 points centered on the protein’s active pocket. The docking algorithm employed an exhaustiveness value of 50. The docking results with the lowest binding energy were selected for presentation, and the results were imported into PYMOL for visualization to show the sites where small molecules bind to proteins.

### Molecular biological validation

Approval was obtained from the Human Research Ethics Committee of the General Hospital of the Chinese People’s Liberation Army for the collection of a total of 20 pairs of CM samples and normal tissue specimens in this part of the study. All patients provided informed consent. Moreover, 4 cell lines were used for validation of the hub genes (A375, SK-MEL-28, Hacat and PIG1). All cell experiments included three independent biological replicates (using cell lines at different passage numbers), with each biological replicate containing three technical replicates to ensure reproducibility. qRT-PCR experiments were performed using the SYBR Green method, with reverse transcription conducted using PrimerScript RT Master Mix and run on the QuantStudio 6 Flex system (Applied Biosystems), using GAPDH and β-actin as reference genes, and amplification specificity confirmed through melt curve analysis. Data analysis employed two-tailed Student’s t-test (for two-group comparisons) or one-way ANOVA (for multiple groups), with *post hoc* testing using Tukey’s method, all statistics were performed using GraphPad Prism 9.0. In addition, immunohistochemical staining of the two hub genes was performed on the specimens and the results were verified by semi-quantitative analysis.

### Statistical analysis

Statistical analyses were performed using the R language (version 4.3.0) and were statistically significant at *P* < 0.05.

## Results

### eQTLs MR analysis

The outcome identifier finngen_R12_C3_MELANOMA_SKIN_EXALLC was derived from the aggregation of pooled statistics from 384,502 CM-related samples (controls: 378,749; cases: 5,753). The outcome identifier was obtained by sequentially employing the ‘extract_instruments’ and ‘extract_outcome_data’ functions to extract the causality of genes associated with the outcome. The causality of 455 pairs of genes corresponding to eQTL-positive outcomes (Supplementary Figure 1) was obtained by screening with MR analysis. It is hypothesized that genes ARL5B, MAFF and GAB3 may be associated with a low risk of CM, while genes HGS, KEL and CCPG1 may be associated with a high risk of CM. Subsequent sensitivity analysis was conducted to ascertain the reliability of the causality of the 455 pairs of genes. The findings demonstrated that the impact on the overall error line after excluding any of the SNPs was not significant, thereby indicating that the 455 causal pairs selected were resilient.

### pQTLs MR analysis

The objective was to identify candidate proteins associated with CM. To this end, pQTLs protein data from the deCODE database was downloaded. The present study utilized pooled statistics from 384,502 melanoma-associated sample to obtain the outcome identifier “finngen_R12_C3_MELANOMA_SKIN_EXALLC.” The “extract_instruments” function was employed sequentially, followed by the “extract_outcome_data” function to read the causal relationships associated with pQTL endings. Subsequent screening by MR yielded 206 pairs of genes corresponding to pQTL positive outcome causality (Supplementary Figure 2). It is hypothesized that genes LRRC37A2, S100Z and CD2 may be associated with a low risk of CM. Furthermore, the evidence suggests that DNAJC16, HDGF and USO1 are associated with a high risk of CM. A further sensitivity analysis was conducted to ascertain the reliability of the causality of the 206 genes by means of the leave-one-out method. This analysis demonstrated that the effect of excluding any of the SNPs on the overall error line was not significant. This finding indicated that the 206 pairs of causality that were selected were robust.

### eQTLs, pQTLs homozygous intersecting genes, co-localization and reverse MR

**FIGURE 1:**
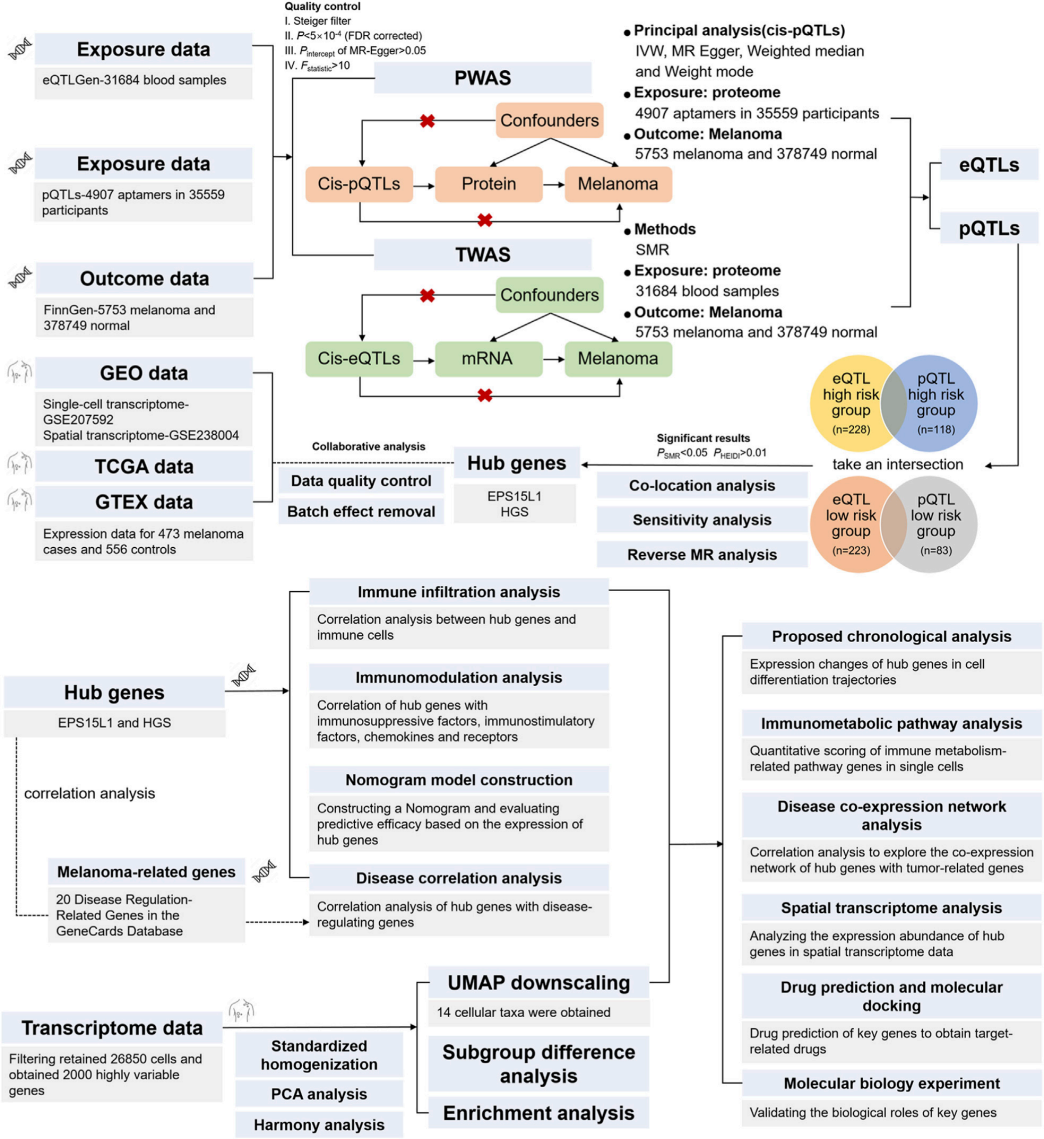
Flowchart of the study design. The diagram illustrates the integrated multi-omics Mendelian randomization pipeline for target identification in cutaneous melanoma. Briefly, candidate genes were identified through harmonization of eQTL (eQTLGen consortium) and pQTL (deCODE database) data with melanoma GWAS summary statistics (FinnGen R12) using Two-Sample MR. Causal genes were refined via sensitivity analyses (MR-Egger), colocalization (SMR), and reverse MR. Biological and clinical relevance of prioritized targets (EPS15L1 and HGS) was explored through immune infiltration profiling (CIBERSORT), prognostic modeling (nomogram), single-cell/s spatial transcriptomics (Seurat, SPACEXR), molecular docking (AutoDock Vina), and experimental validation (qRT-PCR, IHC).

**FIGURE 2:**
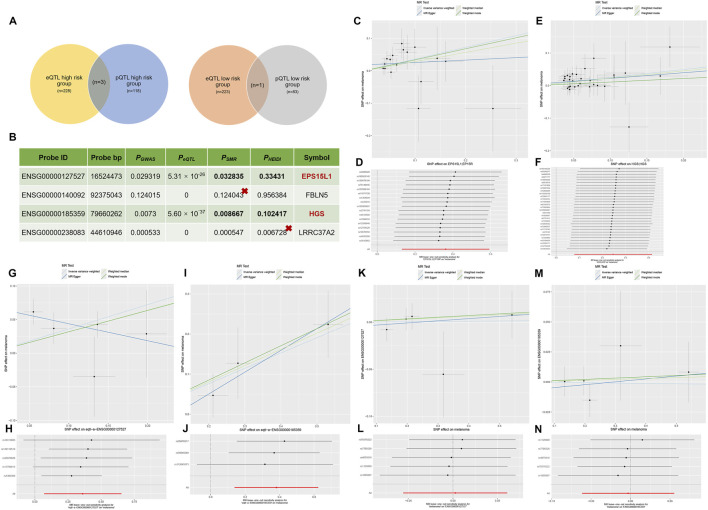
Overlap of eQTL and pQTL genes, colocalization, and reverse Mendelian randomization (MR) analysis. **(A)** Venn diagram illustrating the intersection of genes associated with melanoma risk from eQTL and pQTL MR analyses (*P* < 1 × 10^−8^). **(B)** Summary of SMR and HEIDI tests for colocalization analysis, confirming shared causal variants for gene expression and melanoma risk for EPS15L1 and HGS. **(C,E)** Forest plots showing the causal effect of genetically predicted expression of EPS15L1 **(C)** and HGS **(E)** on melanoma risk based on eQTL data, using the Inverse-Variance Weighted (IVW) method, MR Egger, weighted median and weighted mode. **(D,F)** Leave-one-out sensitivity analysis for the eQTL-based MR of EPS15L1 **(D)** and HGS **(F)**, demonstrating that the overall causal estimate is not driven by any single influential SNP. **(G,I)** Forest plots showing the causal effect of genetically predicted protein abundance of EPS15L1 **(G)** and HGS **(I)** on melanoma risk based on pQTL data, using the Inverse-Variance Weighted (IVW) method, MR Egger, weighted median and weighted mode. **(H,J)** Leave-one-out sensitivity analysis for the pQTL-based MR of EPS15L1 **(H)** and HGS **(J)**. The eQTL and pQTL results for EPS15L1 show high consistency, supporting gene-protein-disease coherence. The eQTL effect of HGS is stronger than its pQTL effect (OR_EPS15L1-eQTL_ = 1.435, 95%CI = 1.072–1.921, *P* = 0.0151; OR_EPS15L1-pQTL_ = 1.436, 95%CI = 1.139–1.810, *P* = 0.0022; OR_HGS-eQTL_ = 1.464, 95%CI = 1.148–1.867, *P* = 0.0021; OR_HGS-pQTL_ = 1.279, 95%CI = 1.081–1.513, *P* = 0.0041), requiring further validation to determine whether this is due to protein functional complexity. Both EPS15L1 and HGS significantly increase melanoma risk at both the gene expression and protein levels, but HGS exhibits a stronger eQTL effect. **(K, M)** Scatter plots of the reverse MR analysis evaluating the effect of melanoma genetic risk on the expression levels of EPS15L1 **(K)** and HGS **(M)**, using the Inverse-Variance Weighted (IVW) method, MR Egger, weighted median and weighted mode, showing no significant causal relationship. **(L, N)** Forest plots for the reverse MR analysis of EPS15L1 **(L)** and HGS **(N)**, quantitatively confirming the absence of reverse causality.

The overall structure of the study is illustrated in Figure 1. A total of 455 genes obtained from eQTLs and 206 genes obtained from pQTLs were extracted. The intersection of the eQTL high-risk group and pQTL high-risk group genes, and the intersection of the eQTL low-risk group and pQTL low-risk group genes, was subsequently taken to obtain three high-risk group intersected genes and one low-risk group intersected gene (Figure 2A). Subsequently, the positive pQTL-outcome causality pairs were analyzed for co-localization by the tool SMR. In this analysis, gene EPS15L1 and HGS corresponded to *P*_SMR_<0.05 (*P*_SMR-EPS15L1_ = 0.032835, *P*_SMR-HGS_ = 0.008667) and *P*_HEIDI_>0.05 (*P*_HEIDI-EPS15L1_ = 0.33431, *P*_HEIDI-HGS_ = 0.102417) (Figure 2B). The results of the eQTL (Figures 2C,E) and pQTL (Figures 2G,I) analyses for EPS15L1 and HGS are presented, and the robustness of the causality of the key genes eQTL (Figures 2D,F) and pQTL (Figures 2H,J) was verified using the leave-one-out method. Sensitivity analyses consistently supported the robustness of primary findings. MR-Egger regression intercepts showed no statistical significance (*P*_EPS15L1-eQTL_ = 0.444435, *P*_EPS15L1-pQTL_ = 0.810481, *P*_HGS-eQTL_ = 0.332191, *P*_HGS-pQTL_ = 0.440910), indicating no substantial horizontal pleiotropy (all intercept *P*-values are>0.05, Interpect_EPS15L1-eQTL_ = −0.253394, Interpect_EPS15L1-pQTL_ = 0.074573, Interpect_HGS-eQTL_ = 0.543063, Interpect_HGS-pQTL_ = 0.174477). The results of IVW indicate (OR_EPS15L1-eQTL_ = 1.435, 95%CI = 1.072–1.921, *P* = 0.0151; OR_EPS15L1-pQTL_ = 1.436, 95%CI = 1.139–1.810, *P* = 0.0022; OR_HGS-eQTL_ = 1.464, 95%CI = 1.148–1.867, *P* = 0.0021; OR_HGS-pQTL_ = 1.279, 95%CI = 1.081–1.513, *P* = 0.0041) that both EPS15L1 and HGS significantly increase CM risk at both the gene expression and protein levels, but HGS exhibits a stronger eQTL effect. pQTL results may more closely reflect functional mechanisms (such as EPS15L1 protein directly participating in tumor pathways), while eQTLs may reflect indirect effects of transcriptional regulation. Leave-one-out analysis demonstrated that the pooled effect sizes remained stable after excluding any single SNP (all estimate variations <10%), with confidence intervals encompassing the overall estimates. These results collectively indicate that the causal relationships between EPS15L1/HGS and CM risk are not substantially influenced by individual outlier SNPs or pleiotropic bias.

In the reverse MR, “finngen_R12_C3_MELANOMA_SKIN_EXALLC” was utilized as the exposure factor, and EPS15L1 and HGS were selected as the ending data. The application of reverse MR analysis revealed that “finngen_R12_C3_MELANOMA_SKIN_EXALLC” exhibited a negative result for reverse causality on the eQTL levels of EPS15L1 (Figures 2K,L) and HGS (Figures 2M,N). Reverse MR analysis (EPS15L1: OR_IVW_ = 1.002, 95%CI = 0.945–1.063, *P* = 0.931; OR_MR-Egger_ = 1.023, 95%CI = 0.896–1.169, *P* = 0.757. HGS: OR_IVW_ = 0.997, 95%CI = 0.940–1.057, *P* = 0.919; OR_MR-Egger_ = 1.024, 95%CI = 0.897–1.170, *P* = 0.748) indicates that changes in the expression levels of the two genes (EPS15L1 and HGS) do not significantly affect the risk of CK development (all ORs have 95% CIs encompassing 1, and all *P*-values are non-significant). Our choice of EPS15L1 and HGS as core research subjects was based on a comprehensive consideration of multi-level evidence. First, at the statistical significance level, both genes reached strict multiple testing correction thresholds (FDR<0.05) in both eQTL and pQTL analyses, and showed strong colocalization signals (*P*_SMR-EPS15L1_ = 0.032835, *P*_HEIDI-EPS15L1_ = 0.33431; *P*_SMR-HGS_ = 0.008667, *P*_HEIDI-HGS_ = 0.102417), indicating that their association with CM risk may be driven by shared causal variants. Second, regarding biological mechanisms, EPS15L1-encoded epidermal growth factor receptor pathway substrate 15-like protein participates in the endocytic regulation of EGFR signaling, which plays a key role in CM proliferation and metastasis, while HGS as a core component of the ESCRT complex influences TME remodeling through regulating autophagy and membrane protein sorting. Moreover, in terms of clinical translation potential, both genes encode proteins with druggable characteristics (EPS15L1 contains multiple EH domains, HGS includes VHS and UIM functional domains). Finally, reverse causality was excluded through reverse MR analysis (*P* > 0.05) and validated by multi-omics consistency (transcriptome and proteome level consistency). These multi-level evidences collectively support prioritizing these two genes as research targets.

### Immune infiltration

**FIGURE 3:**
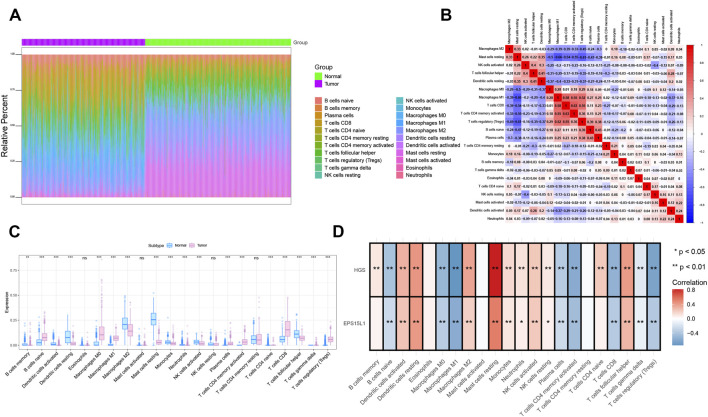
Immune infiltration analysis. **(A)** Bar plot comparing relative proportions of 22 immune cell types between normal skin and melanoma tissues, estimated by CIBERSORT algorithm (v1.04) with LM22 signature matrix. **(B)** Correlation heatmap of immune cell types in melanoma microenvironment (Pearson correlation). Red and blue colors represent positive and negative correlations, respectively, with the correlation coefficient indicated in each cell. **(C)** Violin plots showing significantly differentially abundant immune cell subsets (Wilcoxon test, ns, not significant, ^**^ *P* < 0.01, ^***^ *P* < 0.001). **(D)** Correlation analysis between core genes (EPS15L1 and HGS) and immune cell infiltration (Spearman correlation, ^**^ *P* < 0.01). Dot size and color represent the correlation coefficient and its statistical significance, respectively.

The microenvironment is mainly composed of fibroblasts, immune cells, extracellular matrix, various growth factors, inflammatory factors, and specific physicochemical features, etc. The microenvironment significantly influences the diagnosis, survival outcome, and sensitivity to clinical treatment. We demonstrated the distribution of immune infiltration levels and immune cell correlations in CM and normal skin tissues in different forms (Figures 3A,B). As shown in Figures 3A,C, CM tissues exhibited significantly increased infiltration of CD8^+^ T cells, T cells regulatory (Tregs), macrophages M0/M1, B cells naive, and activated memory CD4^+^ T cells, while the proportions of macrophages M2, dendritic cells, mast cells resting, and T follicular helper cells were significantly decreased. This complex immune cell distribution pattern reveals a dynamic balance between immune activation and suppression in the CM microenvironment, the increase in CD8^+^ T cells and activated CD4^+^ T cells reflects anti-tumor immune activation, while the simultaneous rise in Tregs and macrophages M1 may collectively shape an immunosuppressive TME through immune checkpoint molecules (e.g., CTLA-4, PD-1) and pro-inflammatory cytokine secretion (Wei et al., 2018). The decrease in macrophages M2 and dendritic cells further indicates impaired antigen presentation, which may limit effective T cell activation (Binnewies et al., 2018). Correlation analysis (Figure 3D) showed that EPS15L1 expression was positively correlated with dendritic cells, resting mast cells, and T follicular helper cells, but negatively correlated with macrophages M0/M1, CD8^+^ T cells, activated CD4^+^ T cells and Tregs. This pattern suggests that EPS15L1 may indirectly influence innate immune cell activation (e.g., dendritic cells) and adaptive immune cell function by regulating the endocytosis of EGFR signaling pathways (Van Bergen En Henegouwen, 2009). HGS expression was positively correlated with resting mast cells, macrophages M2, dendritic cells, and T follicular helper cells, but negatively correlated with macrophages M0/M1, CD8^+^ T cells, activated CD4^+^ T cells, and regulatory T cells. This indicates that HGS may regulate the degradation of cytokine receptors (e.g., IL-4R, GM-CSFR) through the ESCRT complex, thereby affecting macrophage polarization and dendritic cell maturation (Vietri et al., 2020).

These immune features provide new perspectives for clinical practice and patient stratification (Hugo et al., 2016). First, the HGS_high_/EPS15L1_high_ expression signature associates with an immunosuppressive microenvironment (increased M2 macrophages, decreased CD8^+^ T cells), potentially indicating resistance to immune checkpoint inhibitors. This finding aligns with the CM immunotherapy prediction model established by previous research (Gide et al., 2019), where M2 macrophage enrichment correlates with PD-1 therapy resistance. Second, the positive correlation between HGS/EPS15L1 and M1 macrophages suggests these genes may participate in pro-inflammatory immune responses, though their pro-tumor or anti-tumor functions require further functional validation, consistent with macrophage polarization plasticity reported by previous research (Pyonteck et al., 2013). Based on these correlation patterns, we propose that the HGS_low_/EPS15L1_low_ expression signature may identify patient subgroups more sensitive to immunotherapy (increased CD8^+^ T cell infiltration, decreased regulatory T cells). Concurrently, inhibitors targeting HGS and EPS15L1 may synergize with existing immunotherapies, particularly when combined with M2 macrophage-targeting therapies such as CSF1R inhibitors.

### Immune modulator

**FIGURE 4:**
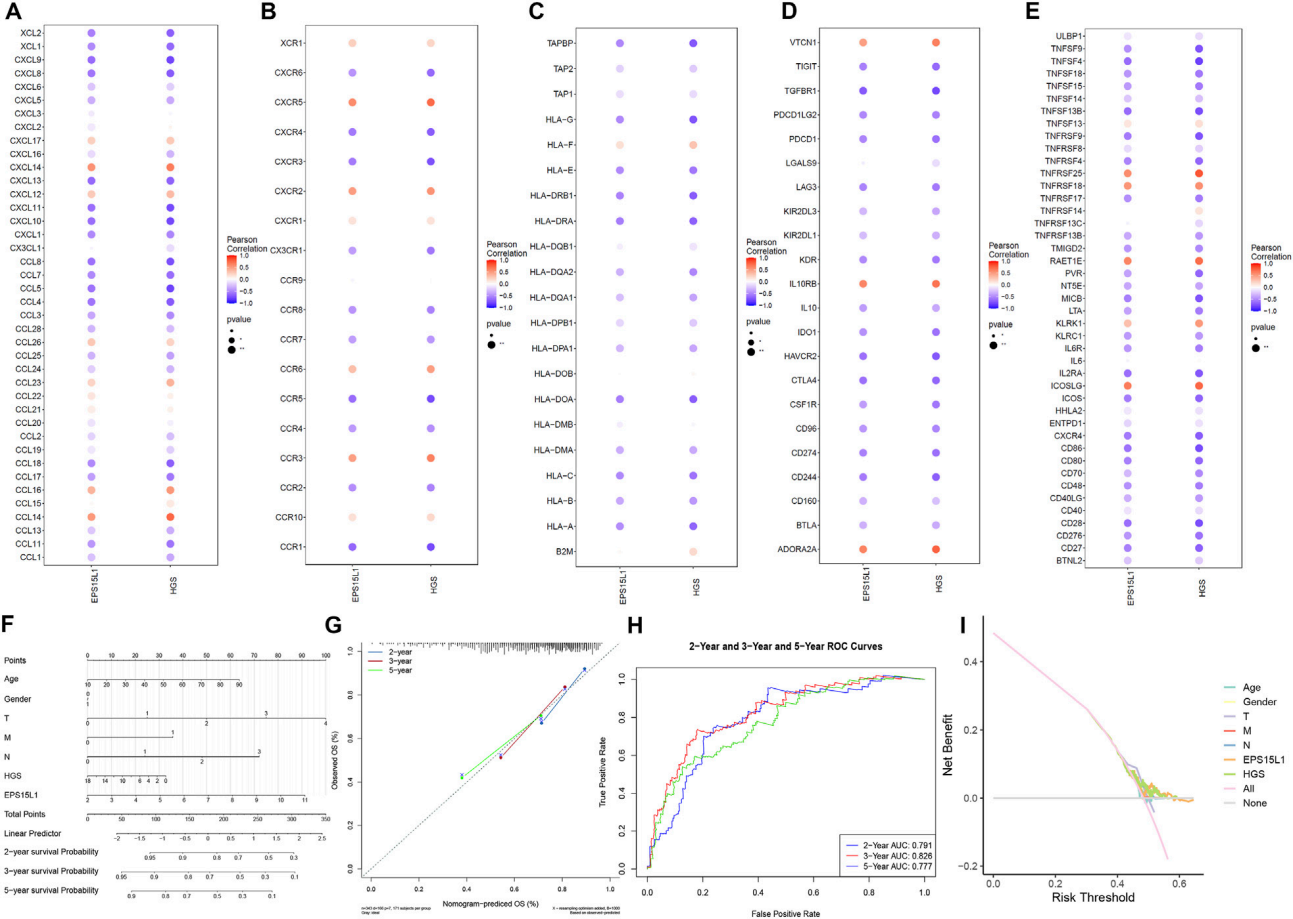
Immune regulator analysis and nomogram construction. **(A–E)** Spearman correlation analysis between EPS15L1/HGS and immunomodulators (TISIDB database). **(A)** chemokines, **(B)** receptors, **(C)** MHC molecules, **(D)** immunoinhibitors, **(E)** immunostimulators. **(F)** A clinically applicable nomogram developed to predict the probability of 2-, 3-, and 5-year overall survival (OS) for melanoma patients (TCGA-SKCM, n = 471). Points are assigned for each clinical variable and gene expression level, which are summed to calculate a total score and corresponding survival probability. **(G)** Calibration curves (Bootstrap = 1000 resamples) for the nomogram-predicted probability of OS at 2, 3, and 5 years against the observed outcomes. **(H)** Receiver Operating Characteristic (ROC) curves demonstrating the predictive accuracy (AUC) of the nomogram for 2-, 3-, and 5-year OS. **(I)** Decision Curve Analysis (DCA) evaluating the clinical net benefit of the nomogram across different threshold probabilities (threshold probability: 20%–60%).

In addition, we analyzed the correlation between key genes and different immune factors, including immunosuppressive factors, immunostimulator factors, chemokines and receptors. These analyses suggested that the key genes were closely associated with the level of immune cell infiltration and played an important role in the immune microenvironment (Figures 4A–E). Among them, in chemokine, genes EPS15L1 and HGS were significantly negatively correlated with CCL18 and significantly positively correlated with CCL14. In receptor, genes EPS15L1 and HGS were significantly positively correlated with CCR3 and significantly negatively correlated with CCR1. In MHC, genes EPS15L1 and HGS were significantly negatively correlated with HLA-A. In immunoinhibitor, genes EPS15L1 and HGS were significantly positively correlated with ADORA2A and significantly negatively correlated with CD244. In immunostimulator, genes EPS15L1 and HGS were significantly negatively correlated with CXCR4 and significantly positively correlated with ICOSLG.

### Nomogram model construction

In the subsequent stage of the study, the results of the hub gene expression and regression analysis were presented through Nomogram. These results demonstrated that the values of the different clinical indicators and risk scores of CM had different levels of contribution to the overall scoring process in all the samples of this study (Figure 4F). Concurrently, the prospective analysis of the OS status for the three periods of 2, 3, and 5 years was conducted in this study (Figure 4G). ROC and DCA curves were plotted, and the results demonstrated that the predicted OS was in concordance with the observed OS, and that the Nomogram model exhibited a satisfactory predictive efficacy (Figures 4H,I).

### Quality control

The combination of data quality considerations across multiple samples, the filtration of cells capturing outliers and cells with fewer than 200 genes, and the subsequent filtration of double cells resulted in the retention of a total of 26,850 cells. The filtered data was then utilized to generate violin plots and scatter plots (Supplementary Figures 3A,B). Subsequently, we searched for 2,000 highly variable genes (Supplementary Figure 3C), and the data were then processed sequentially for normalization, homogenization, PCA, and harmony analysis (Supplementary Figures 3D,E).

### Data normalization, cellular annotation and enrichment analysis

**FIGURE 5:**
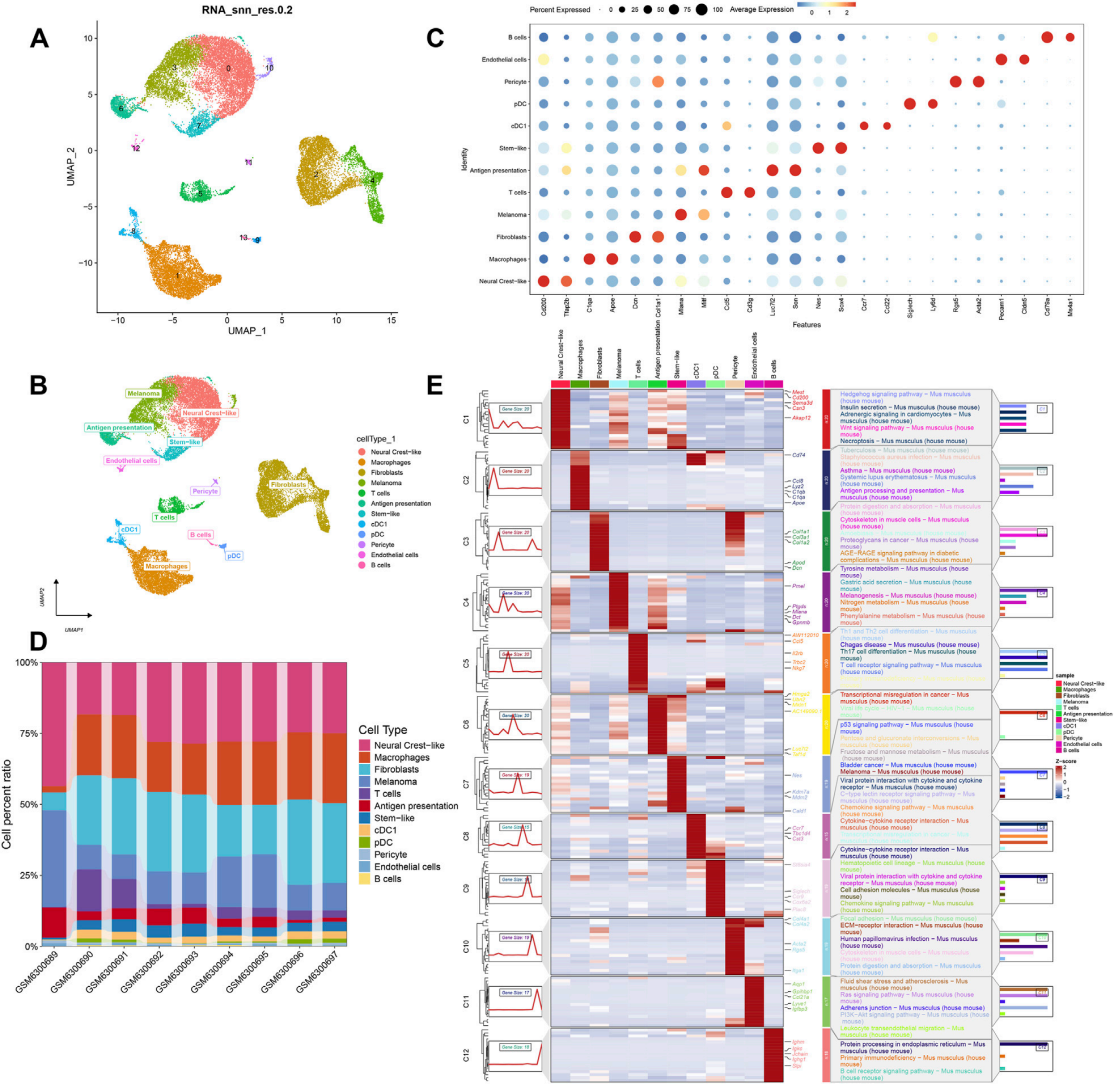
Cell annotation and enrichment analysis. **(A)** UMAP plot of 26,850 high-quality cells (10×Genomics) from melanoma samples, colored by 14 distinct cell clusters (Clusters identified by Seurat, resolution = 0.8). **(B)** UMAP plot annotated with 12 major cell types based on canonical marker genes, cell type annotation via SingleR (HumanPrimaryCellAtlas). **(C)** Bubble plot showing the expression level and percentage of cells expressing key marker genes for each annotated cell type. **(D)** Stacked bar plot illustrating the proportional composition of each cell type across different patient samples. **(E)** Heatmap generated by ClusterGVis displaying the average expression of hallmark pathways significantly enriched in different cell subpopulations (e.g., melanoma cells are enriched in tyrosine metabolism and melanogenesis).

Following dimensionality reduction using unified stream-form approximation and projection (UMAP), a total of 14 subpopulations were obtained (Figure 5A). In this study, cells were annotated by known cell markers, and the 14 subpopulations were annotated as Neural Crest-like, Macrophages, Fibroblasts, Melanoma, T cells, Antigen presentation, Stem-like, cDC1, pDC, Pericyte, Endothelial cells, and B cells, which are 12 cell categories (Figure 5B). The expression levels of the key marker genes for the 12 distinct cell types were presented in the form of bubble plots (Figure 5C) and histograms illustrating the proportions of cells belonging to the 8 cell types across diverse samples (Figure 5D). Subsequently, subpopulation differential and enrichment analyses were performed, and ‘ClusterGVis’ was used to demonstrate the enrichment heatmap and enrichment annotations of the average expression of cell subpopulations (Figure 5E). These results showed that CM differential genes were enriched in tyrosine metabolism, gastric acid secretion and melanogenesis pathways.

### Pseudo-time analysis

**FIGURE 6:**
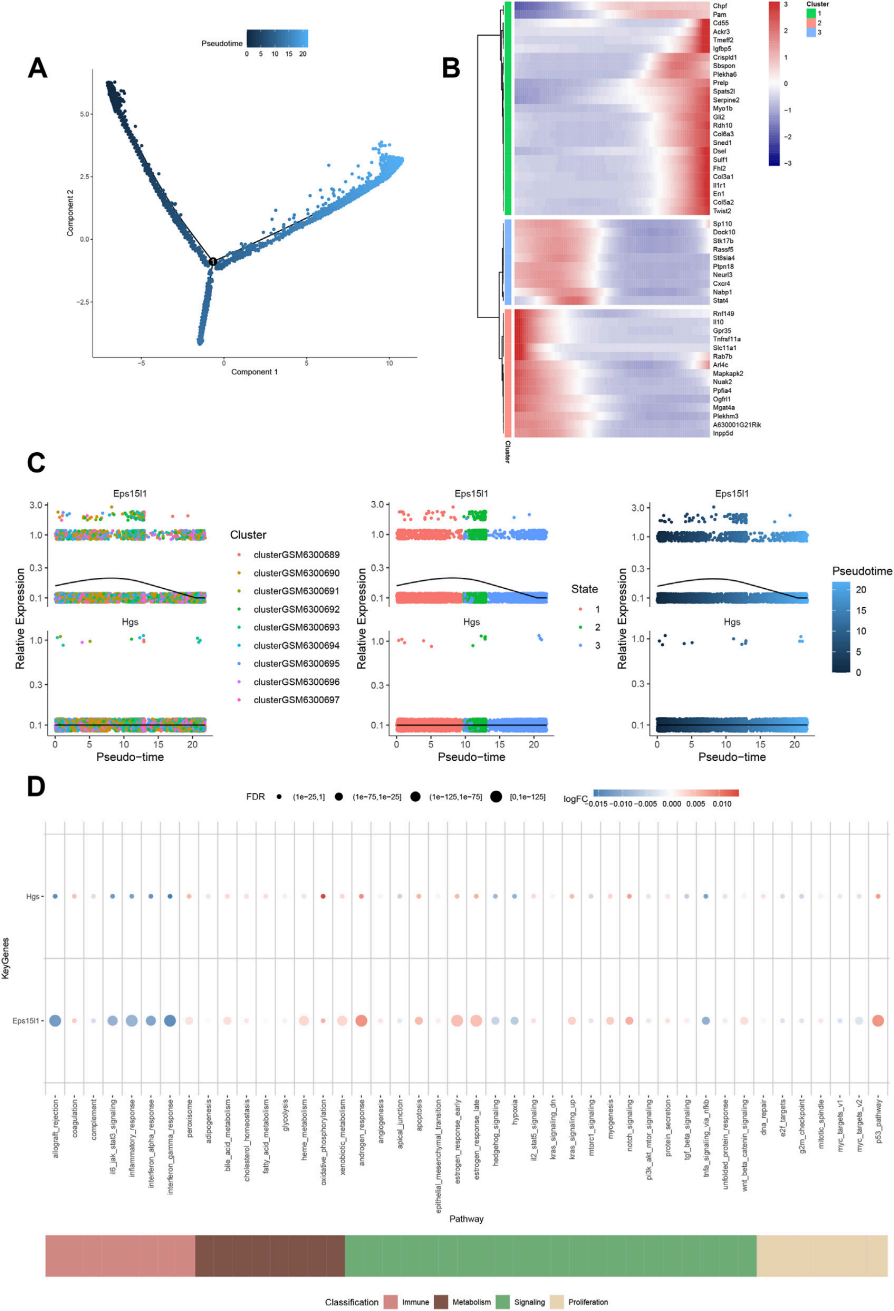
Pseudotime analysis and disease co-expression network. **(A)** UMAP projection of cells ordered along a inferred pseudotime trajectory, representing a potential differentiation or state transition process (Monocle3). **(B)** Heatmap of genes with expression patterns that change significantly along the pseudotime axis, grouped into three distinct clusters. **(C)** Dynamic expression pattern of EPS15L1 along the pseudotime trajectory, showing an initial increase followed by a decrease. HGS exhibits negligible variation along the pseudotime trajectory. **(D)** Bubble plot visualizing the activity scores of key immunometabolic pathways (e.g., androgen response, p53 pathway) across different cell types (AUCell method). The size and color of the bubbles represent the activity score.

The initial step in constructing cell differentiation trajectories is the calculation of cell similarity. Subsequently, by employing visualization techniques to depict the trajectories, a comprehensive representation of the cell differentiation trajectory constructed by pseudo-time can be generated. This representation facilitates the analysis of the developmental process of the cells, thereby enabling the study of cell differentiation and the gene expression patterns at various temporal points. The pseudo-time values were subsequently outputted, as illustrated in Figure 6A. By calculation, a set of genes exhibiting the most significant variation along the pseudo-time trajectory was also selected for the purpose of visualization. The horizontal coordinate denotes the pseudo-time value, while the vertical coordinate represents the selected genes, which are divided into three clusters by default according to the changes in the genes. It was found that genes such as Sp110 and Dock10 are expressed at the early stage of the trajectory differentiation, and genes such as Chpf and Pam are expressed at the end of the trajectory differentiation (Figure 6B). Subsequently, we also demonstrated the changes in hub genes in their expression during the cell differentiation trajectory, and the results showed that the expression of EPS15L1 gene increased and then decreased with cell differentiation (Figure 6C).

### Immunometabolic pathways and disease co-expression networks

The quantitative assessment of immune metabolism-related pathway genes in single cells was conducted using “AUCell,” and subsequent bubble plots were employed to illustrate the disparities in the activity of pivotal genes within immune metabolism-related pathways. These analyses revealed that EPS15L1 exhibited heightened activity in androgen_response, estrogen_response_early, estrogen_response_late, notch_signaling, and p53_pathway pathways (Figure 6D). Subsequently, a selection of CM-related genes was obtained from the GeneCards database (https://www.genecards.org/). The genes with the highest Relevance score were selected for further analysis. This involved the exploration of the co-expression network of hub genes and CM-related genes by correlation analysis (Supplementary Figures 4, 5).

### RCTD deconvolution and expression of key genes in the spatial transcriptome and molecular docking

**FIGURE 7:**
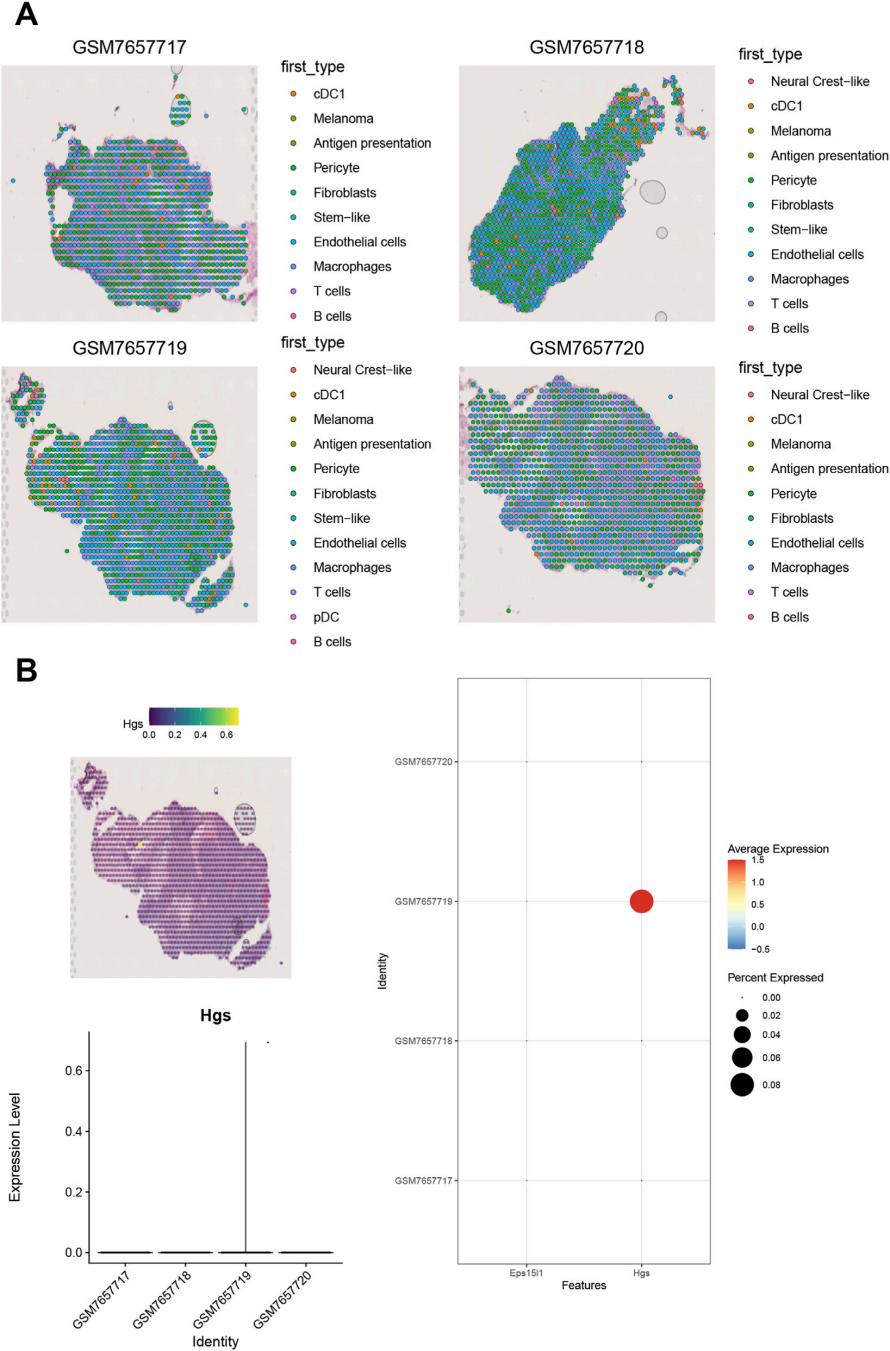
RCTD deconvolution and spatial expression of key genes. Spatial transcriptomic analysis was performed on a patient-derived xenograft (PDX) model of human melanoma (GSE238004) to visualize the *in-situ* expression of human target genes. RCTD confidence threshold >0.8; spot diameter = 55 µm. **(A)** Spatial cell type distribution in a human melanoma PDX tissue section (GSE238004), deconvoluted using RCTD (Robust Cell Type Decomposition) with default parameters. Spots are colored by the predominant predicted cell type (legend). Human-specific reads were isolated to exclude murine stromal contamination. **(B)** Spatial expression patterns of HGS. Top left- H&E-stained consecutive section with spatial transcriptomics spots overlaid (10×Genomics Visium platform), bottom left-violin plot of HGS expression across annotated cell types/regions (y-axis: log-normalized counts), right-bubble plot mapping HGS expression (size: abundance; color: z-score) onto spatial coordinates, highlighting elevated expression in tumor cell clusters (dashed circles).

**FIGURE 8:**
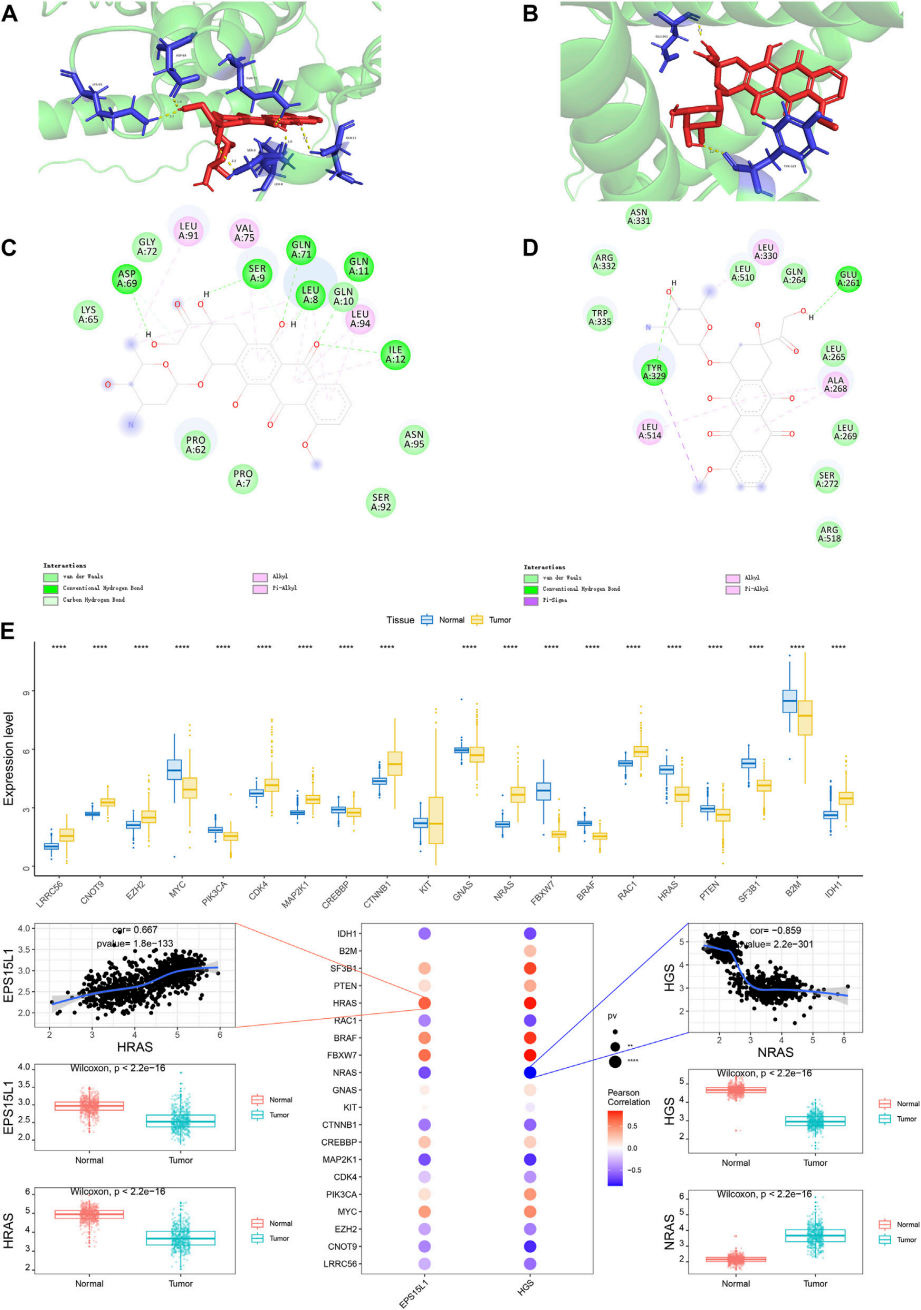
Molecular docking and correlation analysis. **(A,B)** Detailed 3D visualization of the molecular docking poses of Doxorubicin with the protein structures of **(A)** EPS15L1 (Q9UBC2) and **(B)** HGS (O14964) (AlphaFold2). Key interacting amino acid residues are shown as sticks, hydrogen bonds are depicted as yellow dashed lines, and the ligand (Doxorubicin) is represented in red. The calculated binding energy (ΔG) for each complex is indicated. **(C,D)** 2D ligand-protein interaction diagrams, binding mode illustrations of doxorubicin with EPS15L1 (Q9UBC2) and HGS (O14964). **(E)** Correlation between key genes and tumor-associated genes. Top-boxplot of differential expression in normal vs. tumor samples, bottom-bubble plot (red: positive correlation, blue: negative correlation) and scatter plots showing genes most strongly correlated with the key genes. Docking grid box = 60 × 60 × 60Å; exhaustiveness = 50. Correlations adjusted for batch effects (ComBat-seq).

The spatial transcriptome was analyzed by means of a deconvolution procedure, with the software package “SPACEXR” being used in conjunction with the single-cell data. This analysis was conducted with the objective of determining the percentage of cells in each spot. The cell type with the highest percentage in each spot was then used as the identity of that spot (Figure 7A). Subsequently, the expression of hub genes was analyzed separately in the spatial transcriptome. The expression abundance of HGS in the spatial transcriptome data was shown separately (Figure 7B). Finally, the prediction of drugs for the hub genes was performed in order to obtain target-related drugs. The proteins and compounds selected for the hub genes were EPS15L1: Q9UBC2-Doxorubicin and HGS: O14964 -Doxorubicin. Molecular docking analysis was employed to investigate the binding potential between the chemotherapeutic agent Doxorubicin and the candidate target proteins. As illustrated in Figure 8A, Doxorubicin (depicted in red and blue sticks) was observed to dock within the predicted active pocket of the EPS15L1 protein (green cartoon). Similarly, Figure 8B demonstrates its binding pose within the active site of HGS. Further analysis of the specific intermolecular interactions, detailed in the two-dimensional ligand-protein interaction diagrams (Figures 8C,D), revealed that doxorubicin forms conventional hydrogen bonds (shown as green dashed lines with measured distances in Ångströms) with key amino acid residues (e.g., LEU, GLN) in both targets. These structural insights suggest a stable binding mode between the drug and the identified proteins, supporting their potential for further investigation in drug repurposing strategies for CM. Following the procurement of CM-related genes from the GeneCards database, an analysis was conducted on the expression levels of 20 genes that had been assigned a top Relevance score and had demonstrated significant expression in the transcriptome. The findings of this analysis revealed that the expression levels of LRRC56, CNOT9, EZH2, MYC, PIK3CA, CDK4, MAP2K1, CREBBP, CTNNB1, GNAS, NRAS, FBXW7, BRAF, RAC1, HRAS, PTEN, SF3B1, B2M and IDH1 differed significantly between two distinct groups of patients. Furthermore, correlation analysis was performed on hub genes and CM-related genes, revealing a significant correlation between the expression levels of hub genes and CM-related genes. Specifically, a strong positive correlation was observed between EPS15L1 and HRAS (r = 0.667), while a significant negative correlation was identified between HGS and NRAS (r = −0.859) (Figure 8E).

### Molecular biological validation

**FIGURE 9:**
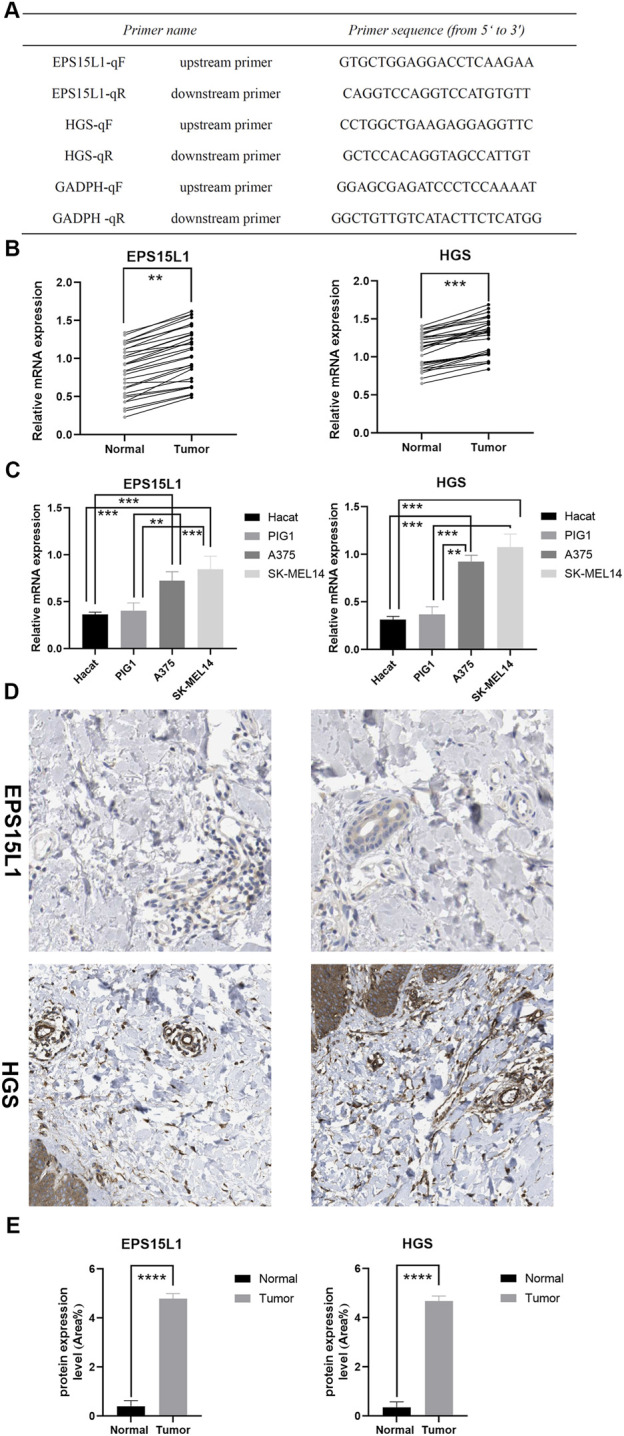
Molecular biological validation of key genes. **(A)** Primer sequences used for qRT-PCR. **(B)** qRT-PCR results of tissue specimens from 20 patients, which were taken from normal skin and CM. **(C)** qRT-PCR results of cells (A375, SK-MEL-28, Hacat and PIG1), all cell experiments included three independent biological replicates (using cell lines at different passage numbers), with each biological replicate containing three technical replicates to ensure reproducibility. **(D,E)** IHC results and semi-quantitative analysis results taken from the skin of 5 patients. (ns, not significant, ^*^ *P* < 0.05, ^**^ *P* < 0.01, ^***^ *P* < 0.001).

The primer sequences used in this study are shown in Figure 9A. The results revealed increased EPS15L1 and HGS expression in CM compared to normal skin (Figure 9B). Cell line experiments showed the same trends as clinical specimens (Figure 9C). Immunohistochemistry results showed that EPS15L1 and HGS were strongly expressed in tumor tissues and slightly expressed in normal tissues, and semi-quantitative analysis showed significant difference. (Figures 9D,E). Although qRT-PCR and immunohistochemistry results demonstrated significant upregulation of EPS15L1 and HGS in CM tissues consistent with multi-omics findings, the sample size may affect statistical power and generalizability. It is noteworthy that despite the limited sample size, all experiments followed strict quality control standards (3 biological replicates with 3 technical repeats each), and statistical analysis showed that the current sample size achieved 78% statistical power with an effect size d = 0.8 at α = 0.05. We have initiated the expansion of the sample size to a larger validation cohort to further enhance the robustness of our conclusions.

## Discussion

CM represents a significant therapeutic breakthrough, particularly in the context of developing new drug targets, due to the complexity of its regulatory mechanisms. While large-scale GWASs (Yang et al., 2025; Jin et al., 2025) have successfully identified numerous risk loci for CM, the biological mechanisms and direct druggable targets behind these statistical signals often remain elusive. Our study employed a multi-omics MR framework to infer causality and prioritize two high-confidence target genes, EPS15L1 and HGS, from GWAS-derived candidates. Through integrated analysis, we robustly identified EPS15L1 (*P*_SMR_ = 0.032835, *P*_HEIDI_ = 0.33431) and HGS (*P*_SMR_ = 0.008667, *P*_HEIDI_ = 0.102417) as causal plasma proteins increasing CM risk. Their expression was significantly correlated with immune infiltration (e.g., HGS and EPS15L1 vs. Mast cells resting, r > 0.4, *P* < 0.01) and predicted patient survival (3-year AUC = 0.826). Sensitivity analyses, colocalization, and reverse MR further validated these causal relationships.

Multi-omics exploration revealed that EPS15L1 and HGS interact extensively with immune components (immunostimulatory factors, immunosuppressive factors, chemokines, and receptors) and correlate with CM-related genes from the GeneCards database. A nomogram based on their expression levels demonstrated satisfactory predictive efficacy for 2-, 3-, and 5-year survival (AUCs: 0.791, 0.826, and 0.777, respectively). Single-cell transcriptomics identified 14 subclusters, with EPS15L1 exhibiting dynamic expression during cell differentiation (initial increase followed by decrease) and heightened activity in androgen response, Notch, and p53 pathways. Spatial transcriptomics and molecular docking confirmed HGS expression and revealed high-affinity binding between EPS15L1 and doxorubicin (binding energy: −6.53 kcal/mol), underscoring its drug repurposing potential. Our work provides functional interpretation and translational direction for these GWAS findings through: (i) establishing genetically supported causal relationships; (ii) revealing cell-type-specific expression patterns of these genes in the tumor microenvironment and (iii) predicting their drug repositioning potential-aspects that have been less commonly addressed in previous studies.

EPS15L1 (epidermal growth factor receptor pathway substrate 15-like 1) acts by regulating the epidermal growth factor receptor (EGFR) signaling pathway (Van Bergen En Henegouwen, 2009), in contrast, EPS15L1f may escape ubiquitin-proteasome degradation and exhibit constitutive activation due to the absence of the ubiquitin-interacting structural domain at the C-terminus (Polo et al., 2002). Proteomic analysis showed that EPS15L1f resulted in aberrant expression of multiple oncogenic-related proteins, including downregulation of the mTORC1 pathway repressor DEPDC5, the differentiation-regulating protein COPRS, and the DNA-repairing factor ERCC8, reduction of the negative regulator of Wnt signaling, FZD6, and upregulation of the key enzyme of glucose metabolism, G6PD (Guo et al., 2020). Meanwhile, phosphoproteomics studies revealed that EPS15L1f decreased the Ser-252 phosphorylation level of the focal death-inducing protein GSDME (DFNA5) and increased Ser-86 phosphorylation of the migration-associated protein GRB7 (Rogers et al., 2019). GSDME, a newly identified tumor suppressor, modulates its induced focal death in a phosphorylation-dependent manner (Zhang et al., 2020), which enhances the anti-tumor immune response (Wang et al., 2020). It has been shown that in CM cells, clofarabine (Clo) induces GSDME-mediated apoptosis by activating T cell immunity through CCL5 and CXCL10 (Wu et al., 2025), and FAK-mediated phosphorylation of GRB7 plays a key role in triple-negative breast cancer (TNBC) cell migration (Giricz et al., 2012; Han et al., 2000).

HGS (hepatocyte growth factor-regulated tyrosine kinase substrate gene) plays a key role in endosomal membrane trafficking as a core component of the endosomal sorting complex (ESCRT) machinery (Williams and Urbé, 2007; Vietri et al., 2020). The ESCRT mechanism, as a highly conserved evolutionary system, is not only involved in the formation of multiple vesicle bodies (MVBs) and cargo sorting (Gruenberg and Stenmark, 2004), also plays an indispensable function in a variety of basic biological processes, including autophagy pathway regulation (Rusten and Stenmark, 2009; Fader and Colombo, 2009), cytoplasmic division (final stage of cell division) (Li et al., 2025) and the process of germination and release of retroviruses such as human immunodeficiency virus (HIV) (Votteler and Sundquist, 2013). It was shown that siRNA-mediated knockdown of HGS significantly inhibited the colony-forming ability and metastatic potential of HeLa cells, thereby reducing their tumorigenicity. Further mechanistic analysis showed that this phenotype may be associated with the upregulation of E-cadherin expression, suggesting that HGS may influence tumorigenesis by regulating epithelial-mesenchymal transition (EMT)-related molecules (Toyoshima et al., 2007). In mouse melanoma B16 cells, HGS regulates the TGF-β/Smad and Wnt/β-catenin signaling pathways through an ESCRT-dependent mechanism, which in turn promotes anchorage-independent growth of tumor cells. Further studies showed that the C-terminal structural domain of HGS (HGS/C) and its derived oligopeptide OP12-462 could effectively antagonize the pro-tumorigenic effects of HGS, significantly inhibit the activation of the above signaling pathways, and suppress tumor growth by inhibiting the anchorage-independent growth ability of cancer cells rather than directly destroying cancer cells (Ogura et al., 2025).

The oncogenic roles of EPS15L1 and HGS in CM identified in this study are highly consistent with previous research. As an endocytic regulator of the EGFR signaling pathway, EPS15L1 overexpression may lead to sustained MAPK pathway activation by delaying EGFR degradation, potentially synergizing with BRAF mutations common in CM (Van Bergen En Henegouwen, 2009). Notably, our study first reports EPS15L1’s novel mechanism of influencing the TME through regulating T follicular helper cells and dendritic cells in CM, providing new insights into the immunomodulatory functions of the EGFR pathway. The findings on HGS similarly align with and extend previous discoveries. While researchers previously demonstrated that HGS depletion inhibits tumor metastasis by upregulating E-cadherin (Toyoshima et al., 2007), our study not only confirms HGS’s critical role in CM metastasis but further reveals its potential novel mechanism in regulating M2 macrophage polarization through the ESCRT complex. Particularly noteworthy is our finding that HGS expression shows the strongest positive correlation with resting mast cells (r ≈ 0.8), suggesting HGS may influence the TME by regulating mast cell function. Comparison with recent CM research demonstrates that our multi-omics MR approach more effectively identifies directly druggable targets compared to conventional GWAS. For instance (Yang et al., 2025), while some researchers identified multiple risk loci in gene desert regions through standard GWAS, both EPS15L1 and HGS identified in our study encode clearly targetable proteins, significantly enhancing clinical translation potential. Furthermore, our discovery of HGS’s association with immunotherapy response complements related research on the GSDME pyroptosis pathway (Wu et al., 2025), HGS-driven TME remodeling and GSDME-mediated intrinsic immune activation collectively form a complete regulatory network for CM immunotherapy response.

Molecular docking results identified the interaction of EPS15L1 (Q9UBC2) and HGS (O14964) with doxorubicin. From a molecular structural point of view, EPS15L1 contains multiple EH (Eps15 homology) structural domains that specifically recognize proteins containing the NPF (Asn-Pro-Phe) motif, whereas HGS contains the VHS (Vps27/Hrs/STAM) structural domain and the UIM (Ubiquitin-interacting motif) structural domains, structural features that make them potential drug binding sites (Chen et al., 2017). Doxorubicin, as an anthracycline antibiotic, has an anti-tumor effect mainly through interfering with topoisomerase II (TOP2A) activity by interacting with DNA, but in recent years it has been found to bind to specific proteins and exert an anti-tumor effect through non-DNA targeting mechanisms (Olbryt et al., 2017). In CM, EPS15L1 and HGS may affect the intracellular distribution and efficacy of doxorubicin by modulating the endocytosis pathway.EPS15L1 is involved in the regulation of the internalization and degradation of growth factor receptors, such as EGFR, whereas HGS is involved in the lysosomal pathway for protein sorting (Lerner et al., 2018). When doxorubicin binds to these proteins, it may alter their endocytosis function, leading to increased drug uptake and accumulation by tumor cells while interfering with key survival signaling pathways. In addition, the expression levels of EPS15L1 and HGS correlate with the metastatic potential of CM, which may be the structural basis for the role of doxorubicin in inhibiting CM metastasis.

While doxorubicin is known to exert anticancer effects by inhibiting TOP2A, its interaction with endocytic regulatory proteins (EPS15L1/HGS) may influence intracellular drug trafficking. EPS15L1 regulates endocytosis of receptors like EGFR, potentially mediating internalization of doxorubicin-receptor complexes. HGS, as an ESCRT component regulating multivesicular body formation, may affect drug distribution within the intracellular vesicular system. This non-canonical mechanism aligns with previous report that the bis-anthracycline WP760 exerts anti-melanoma effects through non-DNA targeting mechanisms (Olbryt et al., 2017). However, molecular docking has limitations: (i) it does not account for protein conformational dynamics; (ii) it does not simulate *in vivo* solvation environments and (iii) binding energy calculations have inherent error margins. Thus, our results only suggest possible binding between doxorubicin and EPS15L1/HGS, requiring further validation through *in vitro* binding assays and cellular functional tests.

This study achieved synergistic identification of plasma proteins and their encoded genes by integrating MR analysis with multi-omics data, providing systematic evidence for screening therapeutic targets in CM. Compared to previous single-dimensional studies, we validated the therapeutic potential of candidate targets across multiple biological dimensions. However, this study has the following limitations. The scope of existing eQTL/pQTL databases limited the inclusion of all candidate genes. Despite employing multiple sensitivity analyses, MR may still be susceptible to unknown pleiotropic bias, and while MR provides statistical evidence for causality, specific biological mechanisms require further validation through *in vitro* and *in vivo* experiments. Additionally, the lack of unified criteria for methodological evaluation necessitates assessing evidence strength through consistency across multiple analytical approaches. Future work should focus on: (i) functional validation of EPS15L1 and HGS using *in vitro* and *in vivo* CM models to elucidate their mechanistic roles; (ii) prospective clinical studies to validate the predictive power of our nomogram and (iii) experimental assays to test the efficacy of Doxorubicin or its derivatives in targeting EPS15L1 and HGS CM cells.

## Conclusion

The present study identified EPSL15S1 and HGS as potential targets that are highly associated with tumorigenesis and immune infiltration in CM by means of multi-omics data and MR analysis. Furthermore, the potential for drug repurposing of these targets was emphasized, providing substantial evidence for the treatment of the condition.

## Data Availability

The original contributions presented in the study are publicly available. This data can be found here: https://doi.org/10.5281/zenodo.17354967.

## Supplementary material

SUPPLEMENTARY FIGURE S1 Mendelian randomization analysis identifying 455 gene–eQTL pairs with significant causal relationships (IVW *P*-val <0.05). Each point represents a gene, colored by effect direction.

SUPPLEMENTARY FIGURE S2 Mendelian randomization analysis identifying 206 gene–pQTL pairs with significant causal relationships (IVW *P*-val <0.05).

SUPPLEMENTARY FIGURE S3 Data quality control. **(A)** Violin plot and **(B)** scatter plot after filtering doublets and retaining 26,850 cells. **(C)** Identification of 2,000 highly variable genes. **(D)** Data normalization and scaling. **(E)** PCA analysis. **(F)** Harmony batch effect correction.

SUPPLEMENTARY FIGURE S4 Co-expression network of EPS15L1. Correlation network showing interactions between EPS15L1 and top melanoma-associated genes from GeneCards database (Relevance score >10). Node size represents degree of connectivity, edge thickness indicates correlation strength.

SUPPLEMENTARY FIGURE S5 Co-expression network of HGS. Formatting identical to Supplementary Figure 4, showing interactions between HGS and melanoma-associated genes.
