# Supplementary material for: Identification of druggable targets in melanoma by multi-omics Mendelian randomization integrated with transcriptomic and spatial analysis
Source: Front Genet. 2025 Oct 27;16:1657356. doi: 10.3389/fgene.2025.1657356 (PMC12597094; doi:10.3389/fgene.2025.1657356)

Symbol;Protein	Nsnp	abs(B)	OR(95%CI)	Pvalue
DNAJC16;DJC16	15	0.4129	1.511(1.192-1.916)	<0.001
HDGF;HDGF	18	0.4118	1.510(1.226-1.858)	<0.001
USO1;USO1	13	0.3713	1.450(1.074-1.957)	0.015
EPS15L1;EP15R	17	0.3617	1.436(1.139-1.810)	0.002
TOLLIP;tollip	15	0.3574	1.430(1.080-1.892)	0.013
DARS2;SYDM	14	0.3447	1.412(1.077-1.850)	0.012
VCL;Vinculin	22	0.3443	1.411(1.133-1.757)	0.002
VTI1B;Vti1_rp1	20	0.3413	1.407(1.189-1.665)	<0.001
SOSTDC1;SOSD1	17	0.3266	1.386(1.113-1.727)	0.004
PSMG3;PSMG3	17	0.3257	1.385(1.098-1.747)	0.006
GGA1;GGA1	12	0.3254	1.385(1.042-1.840)	0.025
NME1;Nucleoside_diphosphate_kinase_A	22	0.3196	1.377(1.121-1.691)	0.002
PIN1;PIN1	17	0.3184	1.375(1.040-1.817)	0.025
CSNK1G2;CSNK1G2	19	0.3142	1.369(1.077-1.740)	0.010
TMOD2;TMOD2	18	0.3069	1.359(1.050-1.759)	0.020
UFC1;UFC1	18	0.3054	1.357(1.058-1.740)	0.016
SARS2;SYSM	18	0.2973	1.346(1.070-1.694)	0.011
HDAC2;HDAC2	25	0.2953	1.343(1.129-1.599)	<0.001
SUMO3;SUMO3	24	0.2933	1.341(1.110-1.620)	0.002
C3;C3	27	0.2924	1.340(1.122-1.600)	0.001
NAA10;NAA10	21	0.2914	1.338(1.064-1.683)	0.013
ACSF2;ACSF2	17	0.2834	1.328(1.020-1.728)	0.035
WWP1;WWP1	20	0.2797	1.323(1.038-1.686)	0.024
RANBP1;RANG	18	0.2767	1.319(1.041-1.671)	0.022
FAM150B;F150B	14	0.2764	1.318(1.015-1.712)	0.038
AGFG1;NUPL	21	0.2747	1.316(1.080-1.603)	0.006
LDLRAP1;ARH	19	0.2744	1.316(1.035-1.673)	0.025
HSF2BP;HSF2B	17	0.2743	1.316(1.009-1.714)	0.042
FMR1;FMR1	22	0.2734	1.314(1.077-1.604)	0.007
NUDT3;NUDT3	25	0.2720	1.313(1.049-1.643)	0.018
BPNT1;BPNT1	18	0.2698	1.310(1.017-1.687)	0.037
LST1;LST1	25	0.2673	1.306(1.134-1.505)	<0.001
FGF20;FGF_20	28	0.2660	1.305(1.125-1.513)	<0.001
LRRC4C;NGL1	18	0.2646	1.303(1.088-1.561)	0.004
KCNIP3;CSEN	20	0.2643	1.303(1.043-1.627)	0.020
ZNF275;ZN275	15	0.2619	1.299(1.023-1.650)	0.032
PGD;6_Phosphogluconate_dehydrogenase	17	0.2604	1.297(1.047-1.608)	0.017
IRF6;IRF6	27	0.2603	1.297(1.034-1.627)	0.024
CIRBP;CIRBP	18	0.2571	1.293(1.046-1.598)	0.017
LAIR2;LAIR2	27	0.2549	1.290(1.038-1.604)	0.022
HOPX;HOP	17	0.2542	1.289(1.068-1.557)	0.008
C3;C3d	17	0.2518	1.286(1.034-1.601)	0.024
GSTK1;GSTK1	15	0.2491	1.283(1.030-1.598)	0.026
DHH;DHH	18	0.2483	1.282(1.003-1.638)	0.047
HGS;HGS	30	0.2461	1.279(1.081-1.513)	0.004
RPE;RPE	20	0.2449	1.278(1.020-1.601)	0.033
RDH16;RDH16	18	0.2422	1.274(1.015-1.599)	0.037
HSD17B7;DHB7	17	0.2419	1.274(1.063-1.526)	0.009
CA2;carbonic_anhydrase_II	17	0.2410	1.273(1.012-1.600)	0.039
UBQLN4;UBQL4	23	0.2406	1.272(1.044-1.550)	0.017
IDO1;INDO	22	0.2398	1.271(1.057-1.529)	0.011
PRPSAP1;KPRA	18	0.2393	1.270(1.025-1.575)	0.029
ELF5;ELF5	18	0.2391	1.270(1.030-1.566)	0.025
CUL3;CUL3	22	0.2368	1.267(1.037-1.548)	0.020
BAG2;BAG_2	25	0.2361	1.266(1.031-1.555)	0.024
MRPL55;RM55	16	0.2346	1.264(1.010-1.583)	0.041
FGF16;FGF_16	21	0.2337	1.263(1.015-1.573)	0.037
ACOT13;ACO13	24	0.2334	1.263(1.029-1.550)	0.026
ATG5;Autophagy_protein_5	26	0.2322	1.261(1.026-1.551)	0.028
KLK15;kallikrein_15	20	0.2320	1.261(1.001-1.588)	0.049
HNRNPF;HNRPF	19	0.2287	1.257(1.013-1.559)	0.037
FYN;FYN	20	0.2282	1.256(1.029-1.534)	0.025
PTPN6;PTP_1C	18	0.2231	1.250(1.071-1.458)	0.005
AMIGO1;AMGO1	18	0.2209	1.247(1.004-1.549)	0.046
SULF2;SULF2	16	0.2169	1.242(1.074-1.437)	0.004
STAT1;STAT1	22	0.2140	1.239(1.012-1.515)	0.038
DBNL;DBNL	26	0.2128	1.237(1.042-1.469)	0.015
PLXNB3;PLXB3	19	0.2125	1.237(1.018-1.503)	0.032
HSP90AB1;HSP_90b	21	0.2125	1.237(1.007-1.519)	0.042
PCNP;PCNP	20	0.2114	1.235(1.005-1.519)	0.045
BLVRA;BIEA	26	0.2096	1.233(1.004-1.515)	0.046
PSIP1;PSIP1	20	0.2057	1.228(1.016-1.485)	0.033
MAPKAPK3;MAPKAPK3	18	0.2035	1.226(1.011-1.486)	0.038
LY6G6C;LY66C	21	0.2002	1.222(1.005-1.484)	0.044
INPP5E;INP5E	19	0.1992	1.220(1.006-1.480)	0.043
BARD1;BARD1	19	0.1983	1.219(1.027-1.447)	0.023
PSMA6;PSA6	24	0.1982	1.219(1.013-1.468)	0.036
DLG2;DLG2	22	0.1965	1.217(1.008-1.469)	0.041
RGS21;RGS21	23	0.1961	1.217(1.006-1.472)	0.044
ADH1C;ADH1G	26	0.1957	1.216(1.010-1.464)	0.039
SH3BGRL2;SH3L2	22	0.1907	1.210(1.019-1.436)	0.029
CD68;CD68	29	0.1905	1.210(1.005-1.456)	0.044
HGF;HGF	26	0.1832	1.201(1.031-1.400)	0.019
SELM;SELM	38	0.1739	1.190(1.021-1.386)	0.026
HN1;HN1	30	0.1735	1.189(1.024-1.382)	0.024
CARS;SYCC	28	0.1699	1.185(1.023-1.373)	0.023
ITGAL;LFA_1_alpha_L_chain	22	0.1682	1.183(1.019-1.374)	0.028
REG3G;REG3G	42	0.1657	1.180(1.034-1.347)	0.014
HNRNPAB;hnRNP_A_B	21	0.1655	1.180(1.028-1.354)	0.018
UBXN2B;UBX2B	17	0.1649	1.179(1.020-1.363)	0.026
FBLN5;fibulin_5	24	0.1646	1.179(1.004-1.384)	0.045
DTX3L;DTX3L	25	0.1637	1.178(1.005-1.381)	0.044
NSFL1C;NSF1C	25	0.1608	1.174(1.022-1.350)	0.024
IBSP;BSP	31	0.1607	1.174(1.026-1.344)	0.020
PSG9;PSG9	27	0.1592	1.173(1.001-1.373)	0.048
CPM;CBPM	33	0.1497	1.161(1.012-1.333)	0.033
MCTS1;MCTS1	29	0.1482	1.160(1.018-1.321)	0.026
ACP5;TrATPase	38	0.1433	1.154(1.037-1.285)	0.009
FKBP1B;FKB1B	26	0.1416	1.152(1.004-1.322)	0.043
GP6;GPVI	31	0.1398	1.150(1.012-1.307)	0.032
CNTN4;Contactin_4	31	0.1374	1.147(1.036-1.270)	0.008
UCMA;UCMA	26	0.1286	1.137(1.005-1.287)	0.041
HS6ST1;H6ST1	33	0.1269	1.135(1.003-1.286)	0.045
SFRP1;SARP_2	28	0.1251	1.133(1.001-1.283)	0.048
ACVR2A;Activin_RIIA	32	0.1221	1.130(1.004-1.272)	0.044
CTSE;CATE	27	0.1183	1.126(1.036-1.223)	0.005
FGFBP3;FGFP3	31	0.1172	1.124(1.004-1.259)	0.042
PLG;Angiostatin	41	0.1163	1.123(1.008-1.252)	0.036
CSAG1;CSAG1	27	0.1147	1.122(1.014-1.241)	0.026
QDPR;DHPR	22	0.1127	1.119(1.015-1.235)	0.025
IGFBP7;IGFBP_7	35	0.1121	1.119(1.014-1.234)	0.025
A1BG;Alpha_1B_glycoprotein	34	0.1093	1.115(1.016-1.225)	0.022
NFASC;NFASC	21	0.1040	1.110(1.011-1.217)	0.028
CCL28;CCL28	51	0.0973	1.102(1.004-1.210)	0.041
CPNE1;CPNE1	30	0.0973	1.102(1.010-1.203)	0.029
GKN2;GKN2	29	0.0967	1.102(1.019-1.191)	0.015
ACP1;PPAC	27	0.0869	1.091(1.011-1.176)	0.024
PNLIPRP1;LIPR1	54	0.0846	1.088(1.004-1.180)	0.041
DDAH1;DDAH1	63	0.0828	1.086(1.007-1.172)	0.032
VEGFA;L_VEGF165	18	0.0810	1.084(1.009-1.166)	0.028
CD33;Siglec_3	15	0.0713	1.074(1.007-1.145)	0.029
KIR2DS2;KI2S2	25	0.0546	1.056(1.006-1.109)	0.029
PLXNC1;PLXC1	29	0.0464	0.955(0.912-0.999)	0.045
NAGLU;NAG	60	0.0523	0.949(0.910-0.990)	0.015
MIA;MIA	34	0.0679	0.934(0.873-1.000)	0.050
GPC1;Glypican_1	37	0.0691	0.933(0.876-0.994)	0.033
SMPD1;ASM	64	0.0698	0.933(0.885-0.983)	0.009
MBL2;MBL	35	0.0701	0.932(0.874-0.995)	0.034
KIR2DL3;KI2L3	26	0.0702	0.932(0.889-0.978)	0.004
SDHAF2;SDHF2	19	0.0745	0.928(0.863-0.998)	0.045
CNRIP1;CB032	27	0.0788	0.924(0.855-0.999)	0.047
GPR68;SPR1	18	0.0821	0.921(0.863-0.983)	0.014
NPFF;NPFF	33	0.0854	0.918(0.859-0.981)	0.011
OBP2B;OBP2B	11	0.0857	0.918(0.855-0.986)	0.019
CREG1;CREG1	40	0.0864	0.917(0.842-0.999)	0.048
UMOD;Uromodulin	41	0.0888	0.915(0.861-0.972)	0.004
NCAM1;NCAM_120	43	0.0899	0.914(0.841-0.994)	0.035
GJA8;CXA8	43	0.0918	0.912(0.850-0.979)	0.010
VWA1;VWA1	37	0.0955	0.909(0.845-0.977)	0.010
UBC;PolyUbiquitin_K63	20	0.0986	0.906(0.849-0.967)	0.003
CEACAM8;CEAM8	52	0.0999	0.905(0.827-0.990)	0.030
ADIPOQ;Adiponectin	48	0.1002	0.905(0.821-0.996)	0.042
TNC;Tenascin	34	0.1009	0.904(0.835-0.978)	0.012
SPARCL1;SPARCL1	44	0.1011	0.904(0.837-0.976)	0.010
FAM174A;TM157	27	0.1015	0.903(0.827-0.988)	0.025
TIMD4;TIM_4	76	0.1078	0.898(0.831-0.970)	0.006
NQO1;NAD_P_H_dehydrogenase	48	0.1164	0.890(0.812-0.976)	0.013
DEFB112;DB112	28	0.1166	0.890(0.795-0.996)	0.043
THBS4;TSP4	28	0.1207	0.886(0.787-0.998)	0.047
ATF6B;ATF6B	32	0.1281	0.880(0.778-0.995)	0.042
POSTN;Periostin	26	0.1291	0.879(0.778-0.993)	0.038
SULT1B1;ST1B1	30	0.1307	0.878(0.794-0.970)	0.010
CD226;CD226	28	0.1328	0.876(0.772-0.993)	0.039
RNF24;RNF24	36	0.1368	0.872(0.792-0.961)	0.006
GLIPR2;GAPR1	28	0.1394	0.870(0.781-0.969)	0.012
FLRT2;FLRT2	34	0.1423	0.867(0.784-0.960)	0.006
FHL1;SLIM_1	23	0.1446	0.865(0.751-0.997)	0.045
AKR1C3;Aldose_reductase_like_C3	24	0.1455	0.865(0.751-0.995)	0.042
ALCAM;ALCAM	28	0.1468	0.863(0.750-0.995)	0.042
IL31;IL_31	30	0.1546	0.857(0.763-0.962)	0.009
CXCL11;I_TAC	47	0.1592	0.853(0.763-0.953)	0.005
PPP1R14A;PP14A	21	0.1633	0.849(0.739-0.976)	0.021
MAGEA6;MAGE_6	27	0.1639	0.849(0.723-0.997)	0.046
APLP1;Amyloid_like_protein_1	27	0.1764	0.838(0.707-0.994)	0.043
IL5;IL_5	33	0.1794	0.836(0.716-0.975)	0.023
ADGRF1;GPR110	18	0.1885	0.828(0.701-0.979)	0.027
PCDHGB1;PCDGD	24	0.1894	0.827(0.694-0.987)	0.035
RS1;XLRS1	22	0.1903	0.827(0.689-0.991)	0.040
PMM2;Phosphomannomutase_2	19	0.1922	0.825(0.689-0.988)	0.036
PRND;PRND	19	0.1940	0.824(0.690-0.982)	0.031
KLK13;kallikrein_13	18	0.1978	0.821(0.690-0.976)	0.025
IHH;ihh	19	0.2007	0.818(0.703-0.953)	0.010
SYNJ2BP;SYJ2B	18	0.2036	0.816(0.675-0.986)	0.035
IL25;IL_17E	17	0.2062	0.814(0.708-0.936)	0.004
HAO1;HAOX1	19	0.2076	0.813(0.669-0.987)	0.036
EGF;EGF	24	0.2079	0.812(0.666-0.991)	0.040
GABBR2;GABR2	26	0.2111	0.810(0.658-0.997)	0.047
LTA;TNF_b	22	0.2136	0.808(0.666-0.979)	0.029
ACLY;ACLY	23	0.2162	0.806(0.662-0.980)	0.031
WDR5;WDR5	21	0.2347	0.791(0.640-0.978)	0.030
CSRP2;CSRP2	17	0.2353	0.790(0.661-0.945)	0.010
SMS;SPSY	20	0.2387	0.788(0.627-0.990)	0.041
TACSTD2;GA733_1_protein	19	0.2415	0.785(0.647-0.953)	0.014
LAT2;NTAL	22	0.2428	0.784(0.638-0.965)	0.022
HMGB2;HMG_2	24	0.2467	0.781(0.633-0.965)	0.022
RGS7;RGS7	18	0.2477	0.781(0.619-0.985)	0.037
PTPN7;PTN7	13	0.2508	0.778(0.609-0.995)	0.045
PSG1;PSG1	22	0.2513	0.778(0.626-0.967)	0.023
RUVBL1;RUVB1	19	0.2532	0.776(0.611-0.986)	0.038
SLC26A5;S26A5	17	0.2536	0.776(0.631-0.954)	0.016
CPLX1;CPLX1	20	0.2540	0.776(0.633-0.950)	0.014
TAGLN3;TAGL3	19	0.2546	0.775(0.618-0.972)	0.027
KCNAB2;KCAB2	29	0.2565	0.774(0.636-0.941)	0.010
ENDOU;PP11	18	0.2604	0.771(0.614-0.967)	0.025
SF3B4;SF3B4	19	0.2628	0.769(0.613-0.965)	0.023
NANS;SIAS	16	0.2679	0.765(0.601-0.973)	0.029
ZPBP2;ZPBP2	17	0.2735	0.761(0.589-0.982)	0.036
FANCF;FANCF	19	0.2766	0.758(0.594-0.968)	0.026
PANK3;PANK3	18	0.2769	0.758(0.603-0.954)	0.018
FEN1;FLAP_endonuclease_1	22	0.2801	0.756(0.598-0.955)	0.019
HECW1;HECW1	24	0.2992	0.741(0.608-0.905)	0.003
CYCS;Cytochrome_c	20	0.3022	0.739(0.557-0.981)	0.036
GDI1;RhoGDI	23	0.3056	0.737(0.584-0.930)	0.010
LRRC37A2;L37A2	17	0.3066	0.736(0.576-0.940)	0.014
S100Z;S100Z	12	0.3191	0.727(0.541-0.976)	0.034
CD2;CD2	21	0.4265	0.653(0.518-0.822)	<0.001

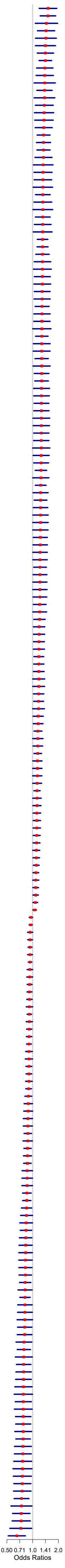

Supplement: Supplementary file 1 [file DataSheet2.pdf]
