# Supplementary figures and images for: Identification of druggable targets in melanoma by multi-omics Mendelian randomization integrated with transcriptomic and spatial analysis
Source: Front Genet. 2025 Oct 27;16:1657356. doi: 10.3389/fgene.2025.1657356 (PMC12597094; doi:10.3389/fgene.2025.1657356)

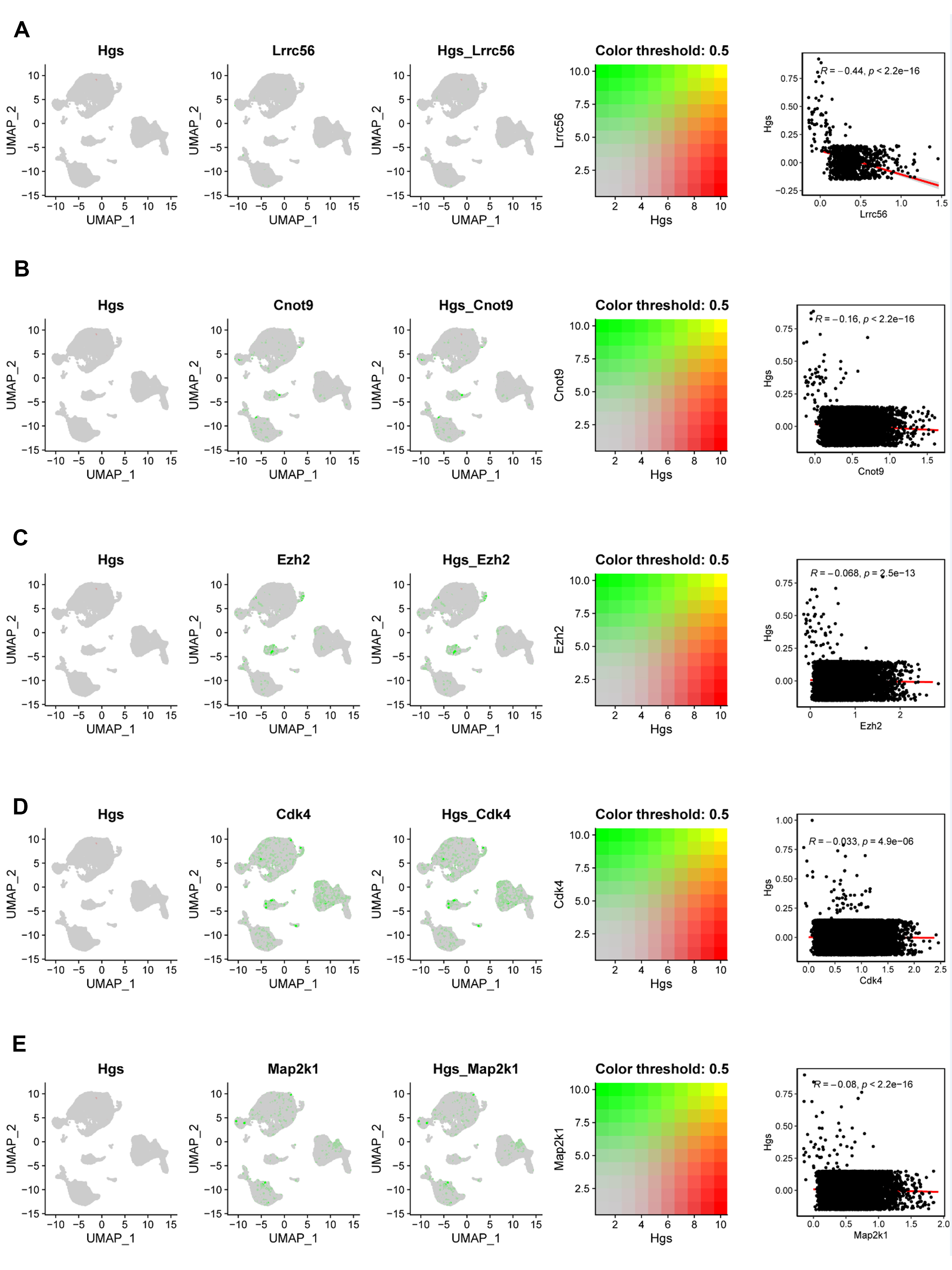

Supplement: Supplementary file 2 [file Image3.jpeg]

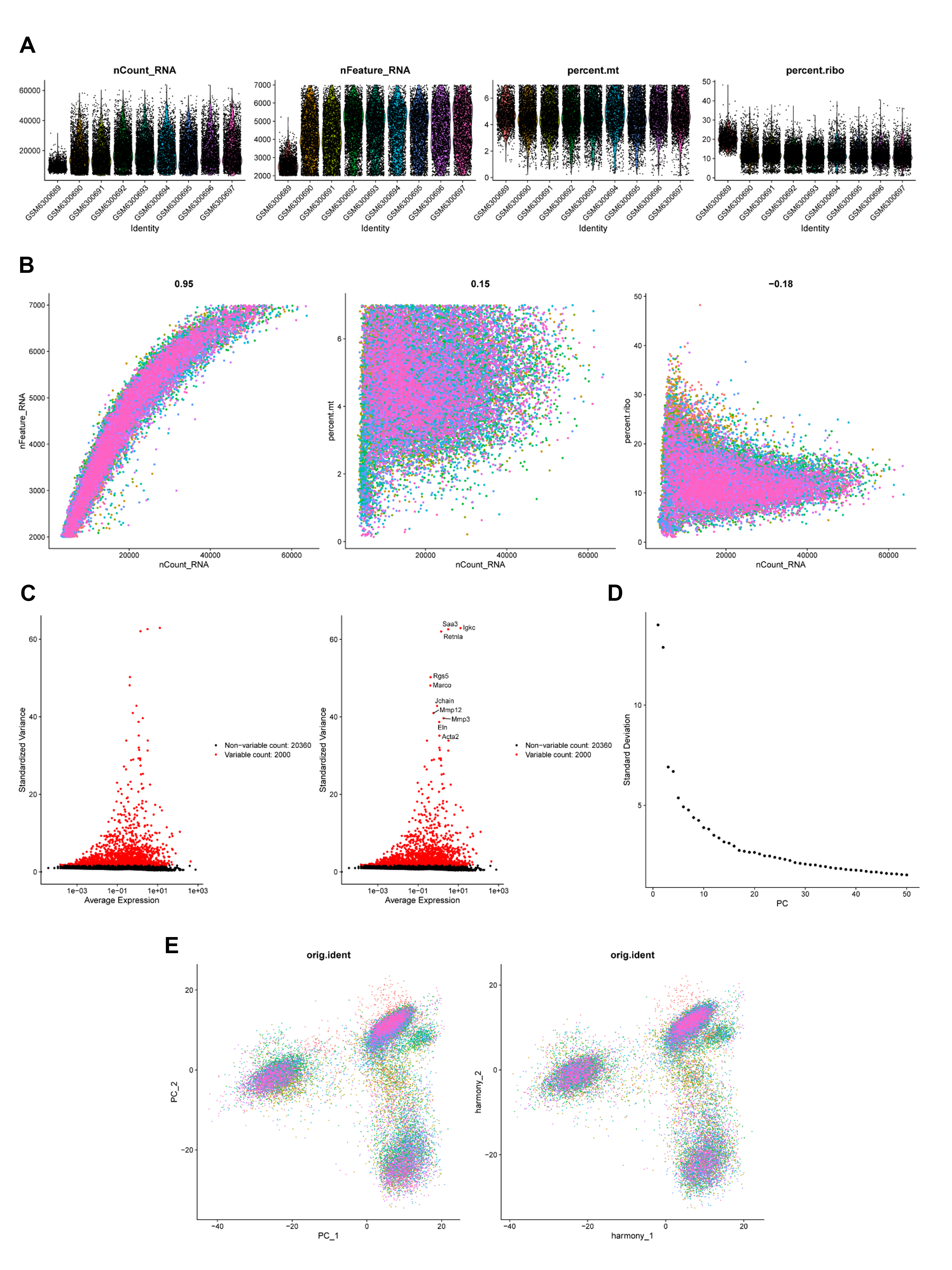

Supplement: Supplementary file 3 [file Image1.jpeg]

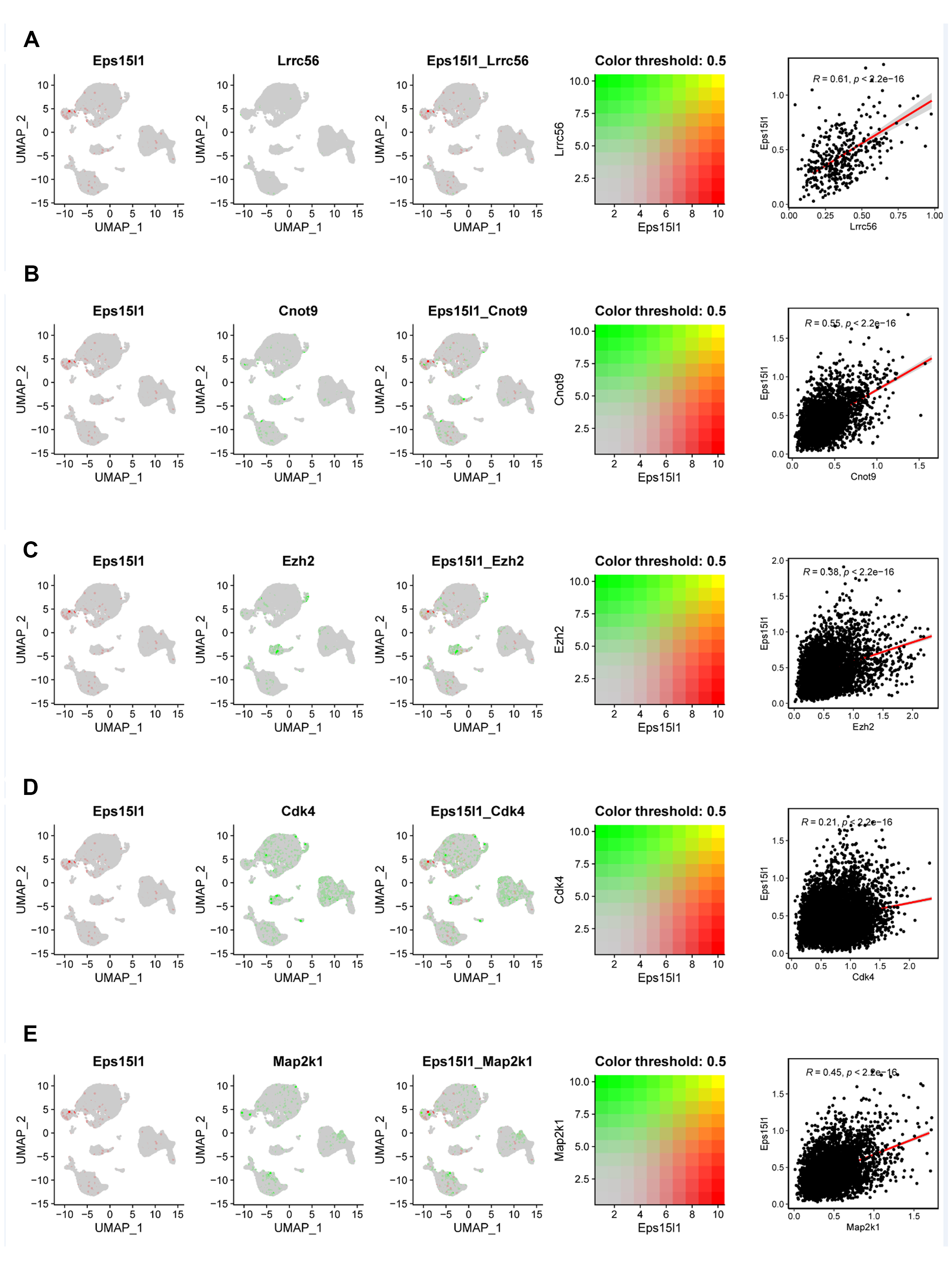

Supplement: Supplementary file 4 [file Image2.jpeg]

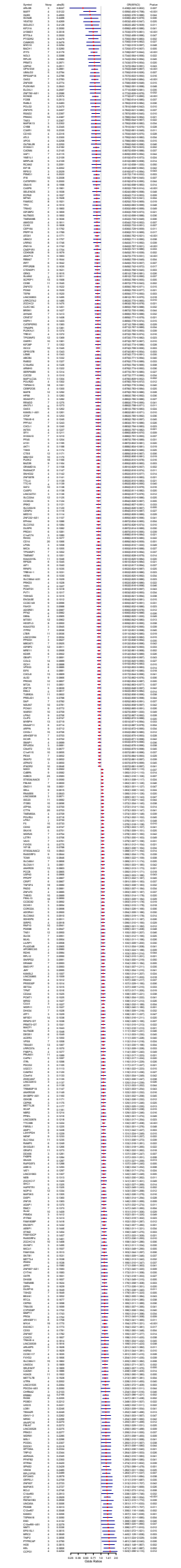

Supplement: Supplementary file 5 [file DataSheet1.pdf]
